# Polymer Chemistry Applications of Cyrene and its Derivative Cygnet 0.0 as Safer Replacements for Polar Aprotic Solvents

**DOI:** 10.1002/cssc.202101125

**Published:** 2021-07-16

**Authors:** Roxana A. Milescu, Anna Zhenova, Marco Vastano, Richard Gammons, Shiliang Lin, Cher Hon Lau, James H. Clark, Con R. McElroy, Alessandro Pellis

**Affiliations:** ^1^ Department of Chemistry Green Chemistry Centre of Excellence University of York, Heslington York YO10 5DD United Kingdom; ^2^ Green Rose, The Catalyst Baird Lane, Heslington York YO10 5GA United Kingdom; ^3^ School of Engineering The University of Edinburgh Robert Stevenson Road Edinburgh EH9 3JL United Kingdom; ^4^ Department of Agrobiotechnology, Institute of Environmental Biotechnology University of Natural Resources and Life Sciences Konrad Lorenz Strasse 20 3430 Tulln an der Donau Austria

**Keywords:** biocatalysis, biomass, enzymatic synthesis, green solvents, membranes

## Abstract

This study explores a binary solvent system composed of biobased Cyrene and its derivative Cygnet 0.0 for application in membrane technology and in biocatalytic synthesis of polyesters. Cygnet‐Cyrene blends could represent viable replacements for toxic polar aprotic solvents. The use of a 50 wt % Cygnet‐Cyrene mixture makes a practical difference in the production of flat sheet membranes by nonsolvent‐induced phase separation. New polymeric membranes from cellulose acetate, polysulfone, and polyimide are manufactured by using Cyrene, Cygnet 0.0, and their blend. The resultant membranes have different morphology when the solvent/mixture and temperature of the casting solution change. Moreover, Cyrene, Cygnet 0.0, and Cygnet‐Cyrene are also explored for substituting diphenyl ether for the biocatalytic synthesis of polyesters. The results indicate that Cygnet 0.0 is a very promising candidate for the enzymatic synthesis of high molecular weight polyesters.

## Graphical abstract

**There once was an ugly duckling…** A binary solvent system composed of biobased Cyrene and its derivative Cygnet 0.0 is investigated for application in membrane technology and in biocatalytic synthesis of polyesters. Cygnet‐Cyrene blends could represent viable replacements for toxic polar aprotic solvents.

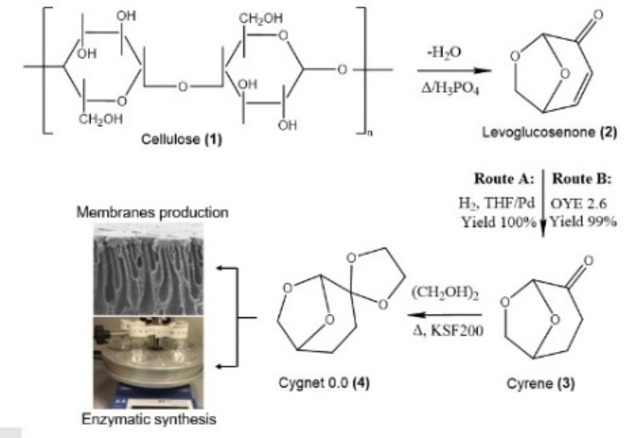

## Introduction

With a multimillion ton annual market and their association with volatile organic compound (VOC) emissions, it is a matter of urgency that solvents need to be manufactured from bio‐derived feedstocks efficiently in order to become sustainable. The majority of solvents are generally synthesized from the major chemical building blocks of the petrochemical industry. They are obtained by the fractional distillation from crude oil and natural gas, followed in some cases by additional solvent extraction, hydrogenation, oxidation, hydration, esterification, methylation or hydrodesulfurization.^[1]^ The solvent typically accounts for 50–80 % of a standard chemical reaction.^[2]^ Under the enormous pressure to deal with challenges and threats of conventional organic solvents which are volatile, highly flammable, toxic and carcinogenic, consideration must be paid to choose the most appropriate alternatives with respect to performance, environmental protection, health and safety impacts and following downstream processing.^[3]^ Ionic liquids (ILs) were considered to be green solvents for more than two decades,[^4^, ^5^] however, commonly used ILs have poor degradability and are toxic, hence they are not considered as green anymore and their use in pharmaceutical and food applications is limited.^[6]^ The conventional polar aprotic solvents require complex syntheses and are associated with several serious problems, and are restricted under the EU legislation on chemicals, Registration, Evaluation, Authorization and Restriction of Chemicals (REACH).[^7^, ^8^] Moreover, they are not easily synthesized from bio‐derived feedstocks and hence, the list of available and safe polar aprotic solvents is currently very small. At present, a series of green alternative solvents to conventional organic solvents have been developed, e. g., bio‐based, waste‐derived, supercritical fluids, natural deep eutectic solvents, etc.[^9^, ^10^] and are listed in Table 1. A new generation of ILs was developed, the deep eutectic solvents (DESs), with higher melting points than of ILs which have been used in the extraction of bio‐active compounds from plants, organic reactions, electrochemistry and enzyme reactions.^[11]^ Moreover, a wide range of solvents are traditionally obtained by fermentation of sugar or starch feedstock,^[12]^ or from anaerobic digestion and wood gasification (bio‐methanol).^[13]^ As for polar aprotic solvents, Cyrene from cellulose waste or *N*,*N’*‐dimethyl‐*N*,*N*’‐dibutylsuccinamide (MBSA) from succinic acid could solve some of the problems associated with the reprotoxicity of the conventional polar aprotic solvents.[^9^, ^14^] As with many new acetal solvents, its peroxide forming potential is much less than the classic ether solvents (e. g., tetrahydrofuran, diethyl ether). Cyrene is known to form a symmetrical aldol condensation product in the presence of a base, as well as being sensitive to strong acids, which limits its use in certain applications.^[15]^ Cyrene has been explored as bio‐based solvent in wire coatings,^[16]^ filtration membranes industry,[^17^, ^18^] pharmaceuticals,^[19]^ graphene dispersion,^[20]^ cross‐coupling,^[21]^ polymers,[^22^, ^23^] MOFs syntheses,^[24]^ solvent extraction^[25]^ or drug delivery^[26]^ where it has replaced toxic polar aprotic solvents such as dichloromethane (DCM), *N*‐methyl‐2‐pyrrolidone (NMP), *N*,*N*’‐dimethylformamide (DMF) or *N*,*N*’‐dimethylacetamide (DMAc).

**Table 1:** Currently available green and bio‐based solvents, including the solvents discussed herein.

Solvent	BP [°C]^[a]^	Applications	Limitations and toxicity profile	Ref.
Cyrene™	227	Platform molecule and solvent replacement for NMP in many applications	Sensitive to strong acids, dimerization in the presence of a base; causes eye irritation	[15]
Cygnet 0.0	285–289^[b]^	Heck reaction and fluorination.	Solid state; miscible in water, which is a concern for aqueous separations	[21]
MBSA	>250	Polymer dissolution, membranes, Heck reaction, MOF synthesis	Not miscible with water, limited solubility of polar molecules	[9]
Water	100	As solvent and reactant, membranes, analysis media	Limited solubility of nonpolar compounds; difficult to burn	[27]
Ethanol	78	Coatings and paint removal, synthetic chemistry, extractions	Highly flammable	[28]
Methanol	65	Coatings and paint removal, synthetic chemistry, extractions	Highly flammable; can lead to blindness	[29]
Ionic liquids	<100	Pharmaceutical, catalysis, biocatalysis, synthetic chemistry, electrochemistry, extractions	Highly viscous, poor degradability and are toxic	[4,30] [6,31]
Deep eutectic solvents	<100	Extractions, biocatalysis, CO_2_ capture, electrochemistry, biomedical applications, catalysis	Highly viscous	[11,32–34]
Supercritical CO_2_		Extraction, polymer production, synthetic chemistry, dry cleaning, semiconductor processing, pharmaceutical industry, coatings	Expensive equipment	[35–37]
Ethylene glycol	196‐198	Platform molecule, materials, biology and medicine	Damages the organs (kidney) in long term	[38]
Lactic acid	122	Platform molecule, food and cosmetic industries	Corrosive, difficult distillation (low volatility)	[39–41]
Glycerol	182	Platform molecule, lubricants, pharmaceutical, food industry	High viscosity	[42,43]
Carbonates	90–243	Platform molecules, solvents, monomers for polymers	Solid state (ethylene c.), flammable (diethyl and dimethyl c.)	[44,45]
Cyclopentanone	130	Membranes, fragrances, pharmaceuticals, pesticides	Flammable	[46,47]
*ɣ*‐Valerolactone	207–208	Fragrances and food industries, membranes, synthetic chemistry	Eye irritation	[48,49]
2‐Methyltetrahydrofuran and 2,5‐Dimethyltetrahydrofuran	78–80	Replacements for tetrahydrofuran (THF) in synthetic chemistry, enzymatic polymerization, pharmaceutical industry	Peroxide forming, unstable in acidic medium, low flash point	[50–52]
2,2,5,5‐Tetramethyloxolane (TMO)	112	Replaces toluene in chemical processes, enzymatic polymerization	Immiscible with water, limited solubility of polar molecules	[53]
Cyclopentyl Methyl Ether	107	Alternative to THF in pharmaceutical industry, synthetic chemistry	Butylated hydroxytoluene (antioxidant) is needed to prevent peroxides formation	[52,54]
Oxymethylene dimethyl ethers (OME_3‐5_)	157‐259	Synthetic chemistry, paint removal, enzymatic polymerization	Immiscible with water, limited solubility of polar molecules	[55]
Triethyl phosphate	215	Membranes, catalysis, plasticizer, stabilizer for peroxides	Causes serious eye irritation	[56]
Dimethyl isosorbide	235	Membranes, drug and permeation enhancer, synthetic chemistry	High boiling point	[57,58]
Dibasic esters	196–225	Paint removal, inks, coatings, adhesives, surfactants	High boiling point	[59]
*D*‐Limonene	176	Platform molecule, clinical applications	Flammable; skin irritation; toxic to aquatic life	[60,61]

[a] B.P. data originated from Meck.co. [a] Taken from Ref. [21].

Cyrene was used as a platform molecule to create other potentially useful compounds. A new bio‐derived compound, Cygnet 0.0, was obtained from the reaction between Cyrene and ethylene glycol and showed promise as replacements for toxic polar aprotic solvents.^[21]^ Cyrene is a bio‐based polar aprotic solvent produced from the hydrogenation of the platform molecule levoglucosenone, which The use of Cygnet as a solvent was previously demonstrated in two pharmaceutical syntheses: Heck reaction and fluorination.^[21]^ In case of a fluorination reaction, Cygnet 0.0 showed similar results as DMF and superior to NMP and acetonitrile, In Heck reaction, both Cygnet 0.0 and Cyrene were comparable to NMP and DMSO.

This work focuses on the prediction and validation of the properties of Cygnet‐Cyrene binary solvent system (Cg−Cy) and the exploitation of Cyrene, Cygnet 0.0, and Cg−Cy as green solvents in membrane preparation and bio‐based polyesters synthesis. Membrane technologies have been widely applied in wastewater reuse and seawater desalination in recent decades, accounting for the largest share of the commercial market for membranes worldwide.^[62]^ The growing use of filtration membranes is the result of increasing attention paid to environmental problems linked to the availability of and growing demand for clean water.[^63^, ^64^] Membrane separation process has many advantages: high water quality, ease of use in clean technology, less energy demand, environmentally benign, greater flexibility in the designing system and easy maintenance.^[65]^

The nonsolvent‐induced phase separation (NIPS) is widely used to prepare membranes and uses large amounts of solvents,^[66]^ generally toxic polar aprotic solvents such as NMP, DMAc and DMF.[^64^, ^67^, ^68^] Therefore, membrane technology attracts increased attention in greener solvents more every year. Sustainable membranes were previously prepared using Cyrene; the viscosity of Cyrene was allowed for and a new method of casting the film from a hot casting gel (70 °C), allowing to tailor membranes to fit a wide range of filtration systems having different physical properties.^[17]^ To our knowledge, Cyrene was not previously used in the preparation of PSF, CA or PI filtration membranes. Cygnet 0.0 and a blend between Cyrene and Cygnet 0.0 were used for the first time in this study in the preparation of all membranes (PES, PSf, CA and PI).

Enzymes have been widely used as catalysts in “green polymer chemistry” due to their benign profile, efficiency in polymerizations, selective reactions under mild conditions and minimal waste generation.[^41^, ^69^] In the past years, there was a renewed interest in utilizing enzymes for the synthesis of functional polyesters that could not be obtained using traditional chemo‐catalysis.^[70]^ More recently, diphenyl ether (DPE) emerged as the election solvent when the enzymatic synthesis of furan‐^[71]^ lignin‐^[72]^ and sugar‐based^[22]^ polyesters was desired since its high boiling point and its polarity characteristics allow 1) the dissolution of a great variety of bio‐based monomers; 2) the feasibility of multi‐day reactions without losing reaction media and 3) easy precipitation of the synthesized polymers with the possibility to recycle the solvent. The few reports on the topic describe the synthesis of chiral epoxides as valuable precursors such as ethyl and methyl (S)‐3‐(oxiran‐2‐yl)propanoates ((S)‐1a/1b)^9^ and the enzymatic reduction of levoglucosenone by an alkene reductase as a sustainable metal‐ and dihydrogen‐free access to Cyrene.^[70]^ Moreover, Cyrene and its diols have only been recently employed as organic media in enzymatically syntheses of polyesters.[^23^, ^73^]

## Results and Discussion

### Characterization of the used solvents

**Scheme 1:**
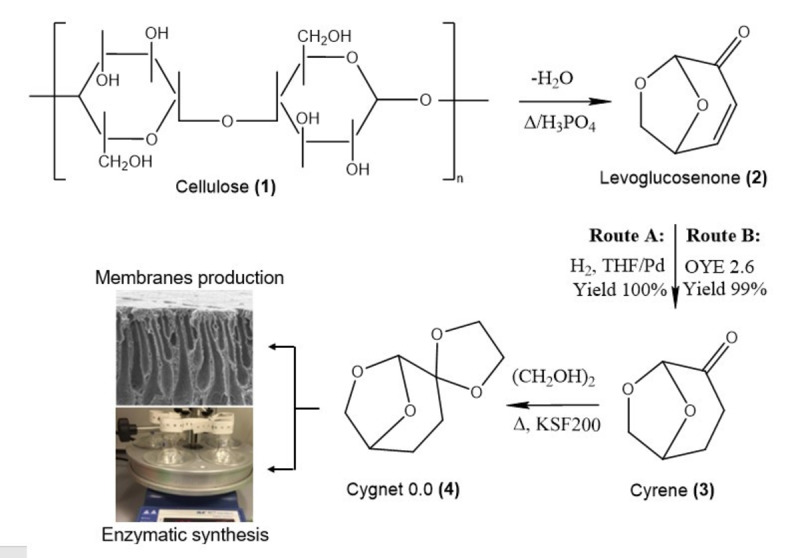
Mechanism of Cyrene (**3**) synthesis[^14^, ^75^] from cellulose (**1**) via levoglucosenone (**2**) and of bio‐derived Cygnet 0.0 (**4**) with the two applications investigated in this work.

The synthesis of Cyrene (**3**) takes only two steps (Scheme 1), whereas that of Cygnet 0.0 (**4**) three steps from the raw feedstock (cellulose). The conversion of levoglucosenone into Cyrene is realized by the hydrogenation of levoglucosenone over a palladium catalyst under mild conditions (Route A in Scheme 1)^[74]^ or through an enzymatic process involving the Old Yellow enzyme 2.6 (OYE 2.6; Route B in Scheme 1).^[75]^ Cygnet 0.0 (**4**) is formed by the reaction of **3** with ethylene glycol in the presence of an acid catalyst (Scheme 1) and has been previously predicted to behave similarly to dichloromethane (DCM).^[21]^

**Figure 1:**
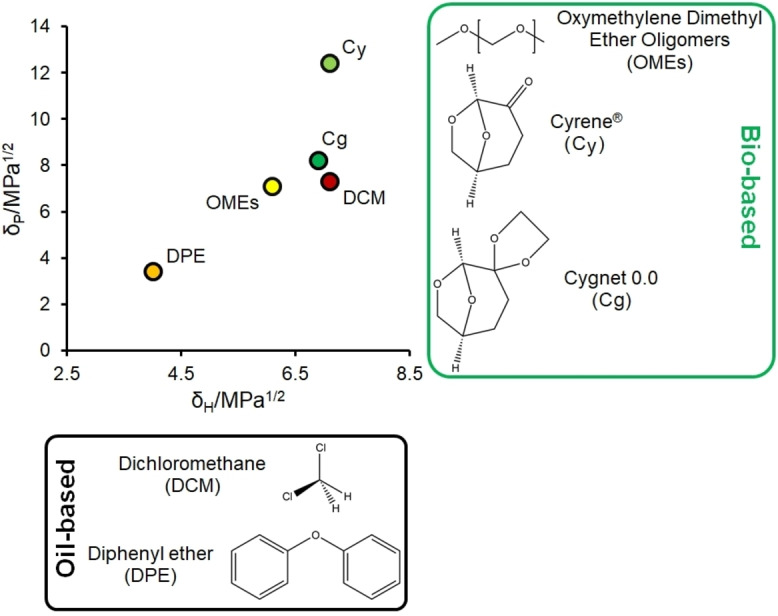
Hansen solubility polarity map including Cyrene (**3**), Cygnet 0.0 (**4**), DCM, DPE and OME_(3–5)_. The complete set of data for the *δ*_P_ and *δ*_H_ calculated values of several solvents of industrial interest are given in Table 2.

Whereas **3** is a liquid with a melting point below −20 °C, making it easy to handle as a solvent under standard conditions, **4** is a needle‐like crystalline solid at room temperature (see the Supporting Information, Figure S1a), with a melting point of 71 °C. By combining **3** with **4**, new binary solvent systems are formed; liquid at room temperature until it reaches a proportion of 50 wt % of **3** in **4**, and more solid with the increase of Cygnet 0.0 concentration (Figure S1). The dynamic viscosity of **3** and Cg−Cy were determined at 25 °C (Figure S2). The viscosity of **4** was not determined due to its crystalline state at room temperature. It was found that the viscosity of a sample with 99.5 % purity of **3** was 0.01162 Pa s (or 11.62 mPa s), whereas for a Cg−Cy solution the dynamic viscosity at 25 C is higher, at 23.07 mPa s. Cygnet 0.0 (**4**) is of particular interest as a solvent, as its Hansen solubility parameters (HSP; Figure 1) are predicted to be very close to those of DCM (Table 2).^[21]^ The polarity parameter, *δ*_P_, is much lower in **4** than in most of the polar aprotic solvents, bringing it closer to DCM (Table 2). This is probably a result of the electronegative ketone moiety being replaced with a five‐membered ring with methylene groups facing outwards, making the molecule overall less polar.

**Table 2:** Predicted HSP for Cyrene and Cygnet 0.0 compared to other solvents with similar properties.

Solvent	*δ*_D_	*δ*_P_	*δ*_H_
Cyrene	18.9	12.4	7.1
Cygnet 0.0	18.3	8.2	6.9
DCM	17.0	7.3	7.1
NMP	18	12.3	7.2
DMSO	18.4	16.4	10.2
DMF	17.4	13.7	11.3
DMAc	16.8	11.5	9.4
Diphenyl ether	19.4	3.4	4
Sulfolane	17.8	17.4	8.7
OMEs 5–5	15.6	7.1	6.1

**Figure 2:**
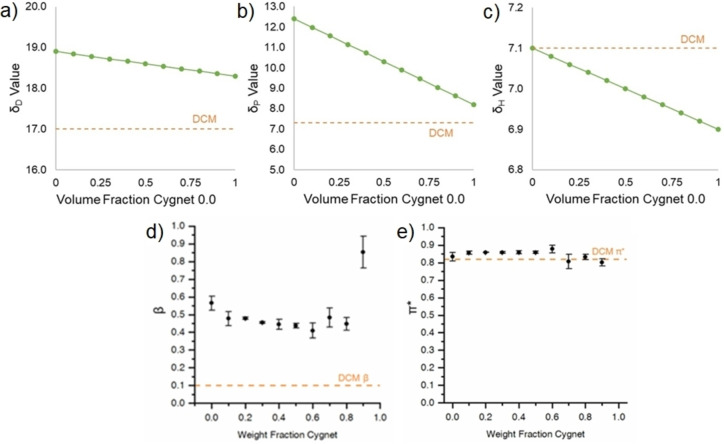
Predicted Hansen dispersion cohesion solubility parameter *δ*_D_ (a), Hansen polar cohesion solubility parameter *δ*_P_ (b), and Hansen hydrogen bonding cohesion solubility parameter *δ*_H_ (c) for Cg−Cy mixtures (continuous dotted green line) versus DCM (dashed orange line). Measured KAT parameters for Cg−Cy mixtures (black dots) vs. DCM (dashed lines): hydrogen bond donating ability *β* (d) and dipolarity/polarizability π* (e). Values are given as averages of three independent trials±standard deviation.

The HSPiP software predicts the solubility parameters of Cygnet‐Cyrene mixtures as a linear combination of the parameters for each individual component (Figure 2). As seen in Figure 2b, the parameter with the most dramatic difference is dipolarity (*δ*_P_), with DCM and **4** being considerably less polar than **3**. Therefore, HSP predicts that to replicate the properties of DCM, the volume fraction of **4** should be maximized to minimize the polarity and dispersion values of the blend. *δ*_D_ (Figure 2a) and *δ*_P_ (Figure 2b) of mixtures move closer to DCM with the increase of Cygnet 0.0 concentration, whereas the opposite is true for *δ*_H_ (Figure 2c). However, the *δ*_H_ values for DCM and **4** are only 0.2 MPa^1/2^ apart, which make them close to each other.

Whereas computationally predicted HSP showed a simple linear trend for each parameter and suggested that maximizing the proportion of **4** gives the best chance of a DCM‐like blend, the experimentally measured Kamlet‐Abboud‐Taft (KAT) solvatochromic parameters are more complex (Figure 2d,e). KAT parameter measurements for Cg−Cy were conducted at 60 °C, based on previously reported methods for measurement of **4** KAT parameters.^[21]^ However, KAT parameters for DCM were measured at 25 °C, due to its low boiling point (39.6 °C). The 90 % Cg−Cy sample was prone to crystallizing during the experiment, explaining the high error margin and possibly the apparent jump in hydrogen bond donating ability. Both *β* and π* show nonlinear relationships with the weight fraction of **4** in **3**. KAT parameters imply the blend closest to DCM is in the range of 50–70 % Cg−Cy, rather than pure **4**. This nonlinear behavior could be the result of complex interactions between **3** and **4** molecules in the bulk.

Only 50 wt % of Cygnet in Cyrene blend was used in this study and it was labelled as Cg−Cy. This mixture has the characteristic of being close to DCM in the HSP solvent space. The viscosity of **3** and Cg−Cy mixtures was also studied here (Figure S2); the determination of the viscosity of **4** was not possible due to its crystalline state. Both **3** and Cg−Cy exhibit a non‐Newtonian behavior, with viscosity decreasing under shear stress.

### Solvent applications

#### Membrane production

The polymers used for membrane fabrication in this study are cellulose acetate (CA), polysulfone (PSf), polyethersulfone (PES) and polyimide (PI), widely used for filtration applications.^[76]^ Cyrene has been previously used to prepare sustainable PES membranes for water treatment and hemodialysis applications.^[17]^ In this study **3**, **4** and Cg−Cy were used to preparing PES, PSf, CA and PI‐based flat sheet membranes, without the use of additives. The cellulose acetate was the first filtration membrane and it is still used for water treatment^[77]^ and hemodialysis^[78]^ and gas separation.^[79]^ Polysulfone (PSf) is used for the fabrication of polymeric membranes^[80]^ with excellent properties such as thermal stability, chemical inertness, mechanical strength and processability.^[81]^ PIs are polymers comprised of imide groups with a stiff aromatic backbone (for thermal stability), chemical resistance, mechanical strength and electrical properties.^[82]^

The results here highlight the importance of combining computational and experimental solubility parameters to find green solvent blends for membranes preparation. 24 solvents were tested in the dissolution of polyethersulfone (PES), one of the most used polymers in membrane fabrication. HSPiP suggested that polar aprotic solvents can be used for dissolving this polymer, results in accordance with prior reports.[^67^, ^83^]

**Figure 3:**
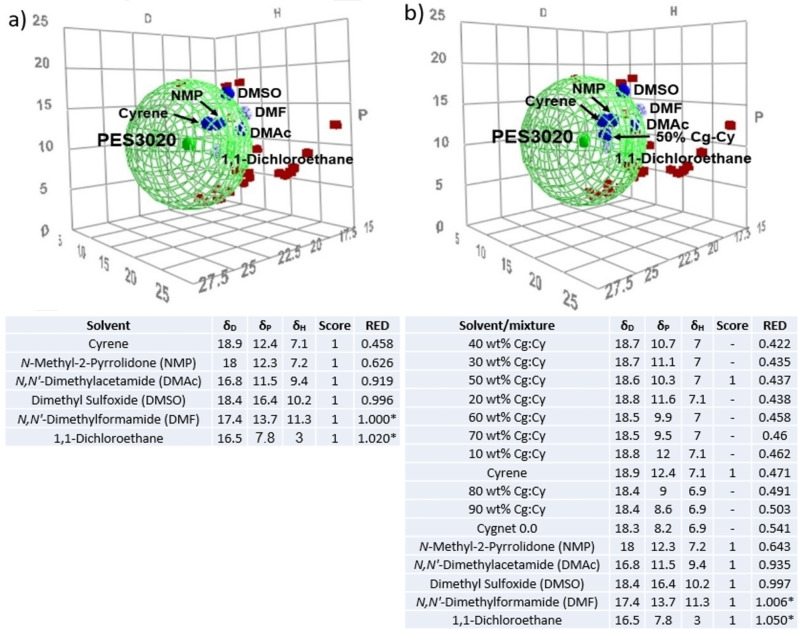
Hansen solubility parameters, the scores given and relative energy distance (RED) of the neat solvents (a) and Cygnet‐Cyrene mixtures (b) proposed to dissolve polyethersulfone PES3020. Only the good solvents (blue spheres) can be seen. „*“ represents solvents wrongly positioned out of the Hansen sphere (they dissolved the polymer).For entries marked with „–“, no score was given (not tested) and RED values were predicted based on the positions of the new solvent systems in Hansen space.

Interestingly, **3** was predicted to be the most suitable solvent in this study, with the smallest value of relative energy distance (RED), hence closer to the polymer core (Figure 3a).^[17]^ Only 50 wt % Cg−Cy was tested in the dissolution of PES and the score “1” was given as a sign of fully dissolving the polymer. However, all Cg−Cy blends were mapped using the parameters of the previously created sphere. The best mixture predicted in this specific case was the 40 wt % Cg−Cy, followed by 30 wt % Cg−Cy and 50 wt % Cg−Cy (Figure 3b). Moreover, the mixtures containing 10 to 70 wt % Cygnet in Cyrene are predicted to be better solvent systems than pure Cyrene (Figure 3b), predicted the best choice for PES polymer in Figure 3a.

**Figure 4:**
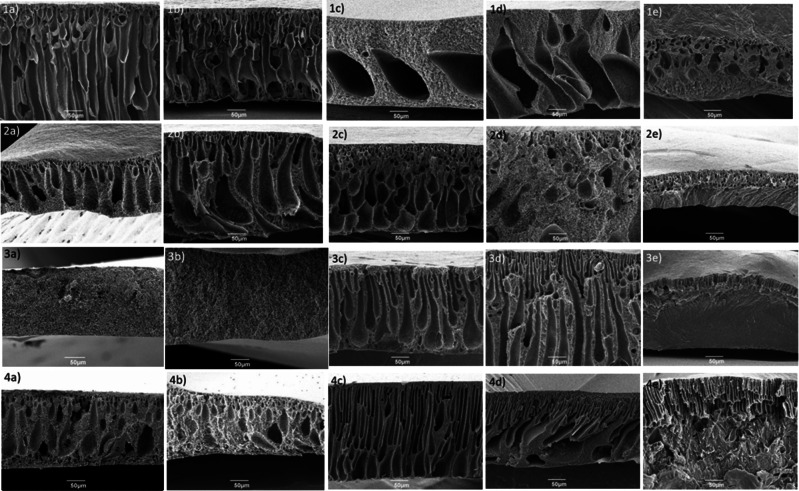
Scanning electronic microscopy images of the cross‐section of membranes produced from cellulose acetate (1), polyethersulfone (2), polysulfone (3), and polyimide (4) and using Cyrene (a,b), Cg−Cy (c, d) and pure Cygnet 0.0 (e) as solvents. The membranes were cast from gels at room temperature (a, c) and from a hot gel at 100 °C (b, d, e).

For ease of handling in both laboratory and industrial settings, the solvents/mixtures used to prepare PES, PSf, CA and PI membranes are pure Cyrene (**3**), pure Cygnet 0.0 (**4**) and 50 wt % Cg−Cy. The scanning electron microscopy (SEM) images of the produced membranes show differences in the membranes morphology when solvent and polymer change (Figure 4). The membranes prepared *via* NIPS process, precipitated after casting due to the immersion in the bath containing water as nonsolvent. Rapid solvent/nonsolvent exchange results in the formation of macro‐voids and finger‐like structures, whereas slow exchange resulted in a sponge‐like or dense structures.^[84]^ The viscosity of a casting solution was found previously to affect the morphology and filtration efficiency of membranes.^[17]^ The solvents used in this study have different viscosities and are expected to affect the morphology of the membranes.

The cellulose acetate (CA) membrane produced using **3** as solvent and cast from a gel at RT or from a hot gel (Figure 4.1a, b) display finger‐like layer through the membrane due to the instantaneous solvent‐nonsolvent de‐mixing during the phase inversion. During the de‐mixing between the solvent (Cyrene) and nonsolvent (water) a mixture of pure water, pure Cyrene and a geminal diol can coexist^[85]^ and are involved in the formation of the porous structures. A Cg−Cy membrane exhibits a completely different morphology when cast from a solution at room temperature (Figure 4.1c); mostly a sponge‐like structure with large macro‐voids, probably due to the air bubbles which did not collapse during the degassing.^[86]^ This sponge‐like layer could resolve the issue of this type of membrane, offering more mechanical strength, often related to high‐pressure filtration applications. In case of CA membrane cast from a hot gel (Figure 4.1d), the viscosity of the solution is lowered with the temperature, hence the de‐mixing happens faster and more macro‐voids can be seen through the thickness of the membrane while less sponge‐like structure was observed.

Polyethersulfone (PES) membranes manufactured by using **3** (Figure 4.2a–e) are typical asymmetrical membranes with Loeb‐Sourirajan structure.^[87]^ Loeb‐Sourirajan membranes present asymmetric pore size and porosity through the thickness of the membrane; the voids are smaller near one surface and bigger on the other surface. The de‐mixing inside the gel happens slower especially towards the bottom layer of the membrane (closest to the glass slide). The membrane prepared using Cg−Cy showcases more non‐interconnected finger layers when cast from a gel at RT (Figure 4.2c) due to the solubility of both **3** and **4** from the mixture Cg−Cy with the ‐solvent (water); no sponge‐like can be seen in this case.

Hot casting gels of PES lead to faster solvent‐water interactions and a faster de‐mixing due to lower viscosity of the gel, generating more macro‐voids and less sponge‐like structures.

Polysulfone membranes produced with **3** (Figure 4a, b) developed a full sponge layer morphology. The temperature of the casting gel does not make a big difference in morphology in this case. As reported in previous studies sponge morphology is associated with greater mechanical strength compared to macrovoidic morphology and is useful for gas filtration.^[86]^ When the mixture Cg−Cy is used, the cross‐sectional morphology obtained is significantly different (Figure 4.3c,d) showing a typical Loeb‐Sourirajan structure with a thin top layer supported on finger‐like layer present through all the membrane thickness and small sponge‐like layer, very similar to PES/Cyrene membranes.

Polyimide (PI) membranes are generally generated from a thermal imidization between a diamine and a dianhydride at high temperature (>250 °C)^[88]^ or a combination between NIPS and imidization leading to sponge‐type polyimide membranes.^[89]^ In this study we produced PI membranes by simply dissolving the thermoplastic polyimide polymer in the solvent, followed by NIPS casting in water (Figure 4.4a–e). When casting polyimide membranes in **3**, the dissolved polymer solution is a less viscous solution with two top active porous layers on top and close to the glass plate (bottom layer). The active porous layer from the bottom layer could be resulted from the fast de‐mixing between the solvent and the nonsolvent which has entered the space between the casting gel and the casting plate, detaching the membrane. Between the two top active porous layers, big finger layers can be seen due to the instantaneous de‐mixing. For a Cg−Cy‐based membrane, the morphology changes. When cast from a gel at room temperature, the de‐mixing is instantaneous, forming non‐interconnected finger‐like layers through the membrane. It was shown before that these non‐interconnected layers could lead to a slow or nonpermeable membrane.^[17]^ When the same membrane is cast from a hot solution, more sponge‐like structure can be seen on the bottom of the membrane, which indicated a slower de‐mixing at the bottom layer (close to the glass plate).

When using pure **4** (Figure 4.1–4.4e) in the casting gel of all polymers, a top active porous layer is formed due to the de‐mixing between the solvent and the anti‐solvent.^[67]^ The porous layer is a mixture of macro‐void and sponge‐like structures in case of CA (Figure 4.1e) and PES (Figure 4.2e) or finger‐like structure for PSf (Figure 4.3e) and PI (Figure 4.4e). The porous layers are supported by a dense layer, bigger in case of PSf and PI, which is seen due to the cooling down of the gel and crystallization of the solvent **4**; no de‐mixing was possible in this layer. This type of morphology was previously shown to have a very small permeability, indicating that it's not suitable for water filtration.^[90]^ The combination of a porous, permeable layer with a nonpermeable layer could be useful for firefighter apparel, sports and military gear similar to a bi‐component polytetrafluoroethylene (PTFE)/polyurethane coatings with waterproof, windproof and heat resistance properties.^[91]^ Sponge‐like membranes give good performance in gas separation or applications where a high pressure is needed (i. e., reverse osmosis).^[92]^ Membranes containing macrovoids are used in water filtration, hemodialysis, food industry or as support in thin‐film composites.[^17^, ^93^]

#### Pure water permeability

**Figure 5:**
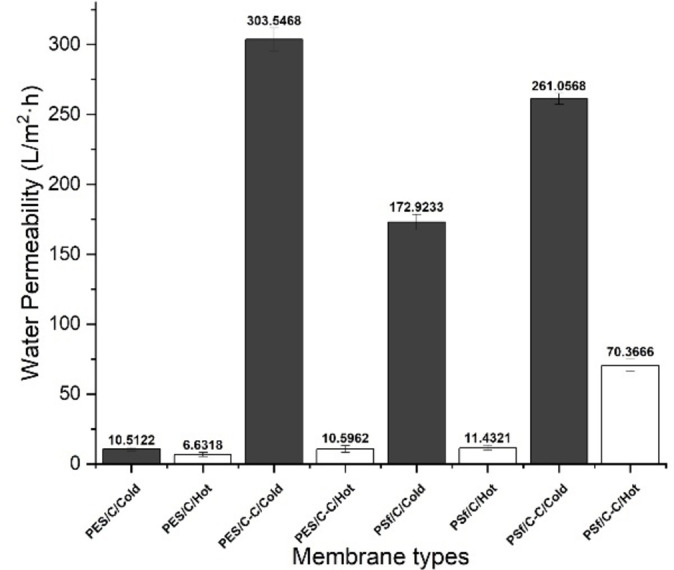
Pure water permeability result for membrane prepared with different solvents and different casting conditions. Hot cast membranes were marked as dark and cold cast membranes were marked as white. Membrane cast with Cyrene marked as C and membrane produced from Cg−Cy marked as C−C.

To explore the effects of membrane cross‐section morphology on its permeability and the practical application of the prepared PES and PSf membranes, their water permeation fluxes were investigated (Figure 5).

In general, both PES and PSf membranes produced with Cg−Cy demonstrated higher flux than the sample prepared with **3**. This indicates that the membranes produced by Cg−Cy are more porous where overall hydraulic resistance is lower. This is consistent with the cross‐section morphology shown in SEM micro graphs (Figure 4). As shown in the cross‐sectional SEM image, using the mixture of Cg−Cy as solvent leads to the formation of more finger‐like structures and less sponge‐like structures. Evidence from literature show that finger‐like layers are more water permeable.[^86^, ^94^] Additionally, hot cast membranes (Figure 4.2a–d and 4.3a–d) are generally thicker than membranes cast at room temperature, which consequently increase the hydraulic resistance,^[95]^ therefore, hot cast PES and PSf membranes demonstrate lower fluxes compared to the cold cast samples.

#### Thermal stability of the produced membranes

Thermogravimetric analysis (TGA) was performed to determine the thermal decomposition of the membranes. The membranes are coded based on the polymer used and the solvent used. For example, a membrane coded as „PSf/Cg−Cy“ will be referred to as polysulfone membrane produces using Cg−Cy mixtures, whereas “CA/Cyrene” is a cellulose acetate membrane produced by using pure Cyrene as solvent. No difference was seen between membranes cast from hot and room temperature casting gels; hence only membranes casted from a gel at room temperature were tested. However, since the membranes manufactured using pure **4** were only cast at 100 °C for this solvent, hot gels membranes were used for TGA. The results of full TGA and differential thermogravimetric (DTG) analysis are given in Figure S3 and summarized in Table 3.

**Table 3:** Thermogravimetric (TGA) and differential thermogravimetric (DTG) measurements of cellulose acetate (CA), polyethersulfone (PES), polysulfone (PSf) and polyimide (PI) membranes.

Sample	*T*_d10_ [°C]^[a]^	Residue [%]^[b]^
CA/Cyrene	365	23.9
CA/Cg–Cy	356	19.1
CA/Cygnet	372	10.1
PES/Cyrene	555	32.8
PES/Cg–Cy	577	33.8
PES/Cygnet	570	41.1
PSf/Cyrene	519	36.2
PSf/Cg–Cy	526	31.5
PSf/Cygnet	528	34.4
PI/Cyrene	> 600	66.7
PI/Cg–Cy	> 600	67.1
PI/Cygnet	518	59.4

[a] *T*_d10_ = Temperature at which 10 % weight loss weas recorded by TGA at 10 °C min^−1^ under nitrogen atmosphere. [b] Weight percentage of material left undecomposed after TGA analysis at 625 °C under nitrogen atmosphere.

As seen in Table 3, the most thermally stable membranes are produced from polyimide, with high decomposition temperature (*T*_d_=518–600 °C), whereas cellulose acetate‐based membranes have the lowest decomposition temperatures (between 355–372 °C). Thermal decomposition occurs for **3** at 165 °C while **4** decomposes at 210 °C (Figure S3.5a, b). No residues can be seen after carbonization of **3** under a flow of nitrogen, whereas the degradation of **4** resulted in 8.8 % residue.

Cg−Cy mixture produced PES an PSf membranes with high decomposition temperatures of 577.1 and 526.1 °C respectively, higher than **3**‐based membranes, due to the higher thermal stability of **4** from the mixture. In case of CA, **4** produced the highest thermally stable membranes, with thermal decomposition at 371.9 °C. The PI membranes decompose at high temperature, at around 600 °C (the interval RT‐625 °C was not enough to measure the *T*_d_ accurately in these cases Figure S3.4b) but have a higher thermal stability than PI‐based membrane produced using **4** (518.2 °C). CA‐based membranes produced the smallest residual material, whereas PI‐based the highest yield (over 59 %). This means that PI membranes are most thermally stable, whereas CA‐based filtration membranes have the lowest thermal stability.

#### Biocatalytic polycondensation reactions

After the formation of membranes, another tested application of **3** and its derivative **4** was their utilization as solvents for the enzymatic synthesis of polyesters with the aim of substituting DPE, current election solvent for these polycondensation reactions.

Following our previous work on the utilization of oxymethylene dimethyl ether oligomers (OME)^16^ and its comparison with DPE, we initially selected to perform a similar set of reactions using dimethyl adipate (DMA) as the diester and 1,4‐butanediol (BDO, C_4_) and 1,8‐octanediol (ODO, C_8_) as the aliphatic diols.

**Figure 6:**
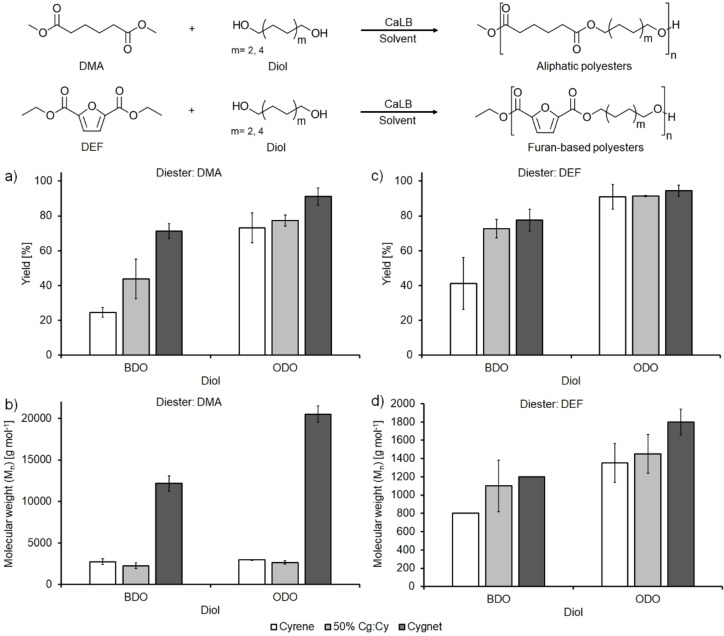
Enzymatic synthesis of aliphatic and furan‐based polyesters. Reaction yield after the three purification steps for a) aliphatic and c) furan‐based polyesters and number average molecular weight (M_n_) of the synthesized polymers (calculated by GPC) for b) aliphatic and d) furan‐based polyesters. BDO=1,4‐butanediol (C_4_); ODO=1,8‐octanediol (C_8_).

The collected data show that increasing the carbon chain length of the diol from C_4_ (BDO) to C_8_ (ODO) when using pure **3** (Figure 6a, white bars) or a 50 % Cg−Cy mixture (Figure 6a, light gray bars) as the solvent, leads to an increase of the reaction's yield from 25 % and 44 % to 73 % and 77 % respectively. For the reaction carried out in pure **4** (Figure 6a, dark gray bars), the increase is less evident since there is only a 17 % yield increase (from 74 % to 91 %). The yields are in correlation with the molecular weight distribution data plotted in Figure 6b.

From these data, it is possible to observe how the reactions carried out in the more polar solvents (**3** and 50 % Cg−Cy) led to oligomers having limited molecular weights (*M*_n_=2300–3000 g mol^−1^, *M*_w_=5800–7900 g mol^−1^) while the reactions in pure **4** led to polymers having *M*_n_/*M*_w_= 12200/31400 g mol^−1^ for C_4_ and 20500/35300 g mol^−1^ for C_8_.

The collected data fit the trends observed for the synthesis of the same adipate‐based polymers in OMEs and DPE. Again the most successful polymers synthesized were the ODO‐based ones, reaching *M*_n_/*M*_w_ of 7400/9700 g mol^−1^ and 10400/13600 g mol^−1^ when the syntheses were carried out in OMEs and DPE, respectively.^[55]^ The difference in the recovered yields is most probably due to shorter polymer chains being more soluble in the nonsolvent (ice‐cold methanol) and therefore are lost during the precipitation and washing steps. This observation, together with the lower obtained molecular weights, is consistent with the reactions between dimethyl adipate and various diols that were carried out in a solvent‐less reaction system; in fact, also, in this case, the C_4_ BDO led to polymers having slightly lower molecular weights (*M*_n_/*M*_w_=6600/11500 g mol^−1^) in comparison with the reactions carried out using the C_6_ and C_8_ diols (*M*_n_/*M*_w_=6700/13700 g mol^−1^ for C_6_ and 7100/12600 g mol^−1^ for C_8_, respectively).^[96]^ To extend the scope of the work and to investigate the synthesis of other bio‐based polymers, we also carried out the same set of reactions substituting dimethyl adipate with diethyl‐2,5‐furanoate (DEF) as the diester to prepare aliphatic‐aromatic polyesters. In this case, all yields were higher in comparison to the adipate‐based polymers since the solubility of the short furan‐based oligomers in MeOH is significantly lower than the one of the aliphatic polymers due to their aromatic character. The data shows a similar trend with the increase of the recovered yield with the increase of the diol's carbon chain length from C_4_ to C_8_ (Figure 6c). A similar increase was also observed for the molecular weight distribution (Figure 6d) but in this case, despite excellent monomers conversions >93 % (as calculated *via* ^1^H NMR), only short oligomers were recovered (800/1000 g mol^−1^<*M*_n_/*M*_w_<1800/3000 g mol^−1^). The obtained limited molecular weights are in line with previously reported polymers synthesized using various conditions (time, solvent and vacuum but all using CaLB as the catalyst). In particular, an *M*_n_ of roughly 2300 g mol^−1^ was attained when using 4‐(2‐hydroxyethoxy)benzoic methyl ester as the monomer while 1100 g mol^−1^<*M*_n_<2400 g mol^−1^ were obtained by a previous work using dimethyl‐2,5‐furandicarboxylate as the diester and BDO and ODO as the aliphatic diols.[^71^, ^97^]

An additional explanation for why polymerizations of BDO in **3** containing systems resulted in both lower molecular weight and yield is the potential for side reactions between the solvent and the diol.

Further analysis of the obtained materials by ^1^H NMR spectroscopy and MALDI‐TOF mass spectrometry revealed that, when **3** was used as the organic media in combination with either of the two diols, the solvent somehow became incorporated in the polymer chain, leading to a variety of new signals in the ^1^H NMR and MALDI spectra (Figures S4–S7). Moreover, when using the 50 % Cg−Cy mix, **3** was again incorporated in the polymer chain, albeit to a lesser extent (Figures S8 and S9). When using **4** as the media, no side reactions were observed, with the polymer presenting a MALDI distribution typical of polyesters with the various end groups clearly visible (Figures S10–S13). These observations fit very well with observations reported by Vastano et al. when polymerizing the multifunctional galactaric acid with BDO. In fact, in this previous paper, the authors observed that **3**, when used as the organic media for catalyzing enzymatic transesterification reactions, was able to form intra (between the secondary hydroxy groups of mucic acid) and trans‐chain (between BDO and mucic acid) ketals, with the subsequent loss of the polymer's hydroxy functionality.

#### Thermal analysis of the synthesized polymers

The thermal analysis of the synthesized polymers follows very well the trends that we discussed for the GPC results (Table S2). The aliphatic polymers synthesized from DMA and the C4 diol, 1,4‐butanediol show an increasing *T*_d10_ temperature going from 359 °C to 361 °C and to 364 °C when **3**, 50 % Cg−Cy and **4** are used, respectively. Similarly, when using the C_8_ diol 1,8‐octanediol in combination with the same diester, the temperatures increase from 369 °C to 372 °C and to 376 °C when **3**, 50 % Cg−Cy, and **4**, respectively, are used as solvent. From the DSC analysis, we can observe that all polymers show a crystalline behavior showing a *T*_c_ and a *T*_m_. Also, in this case, an increase of *T*_c_ and *T*_m_ is significant when changing the reaction's solvent, in particular when using BDO as the diol the *T*_c_ and *T*_m_ increase from *T*_c_=26 °C/*T*_m_=43 °C to *T*_c_=32 °C/*T*_m1_=48 °C‐*T*_m2_=50 °C and to *T*_c_=35 °C/*T*_m1_=49 °C‐*T*_m2_=55 °C while when using ODO as the diol the following trend is observed: *T*_c_=46 °C/*T*_m_=61 °C to *T*_c_=42 °C/*T*_m_=61 °C and to *T*_c_=51 °C/*T*_m1_=66 °C when using **3**, Cg−Cy and **4** as the solvent, respectively (Figure S14).

## Conclusion

The bio‐based polar aprotic solvent Cyrene (**3**) was used in this study as a solvent and precursor for other solvent/solvent systems further used in applications such as membrane science and synthesis of polyesters. Simply blending **3** with its derivative Cygnet 0.0 (**4**) led to the formation of new solvent systems with different properties. In situ synthesis of Cg−Cy facilitates the use of the new solvent system in applications where the purification of **4** is not necessary. In this work, **4** and a 50 % Cg−Cy mixture were used for the first time in membrane preparation and enzymatic synthesis. Polyimide, polysulfone, polyethersulfone, and cellulose acetate‐based flat sheet membranes for filtration applications were prepared by NIPS technique by using the bio‐based solvent **3**, its derivative **4**, and a mixture of the two. The membranes were produced without the aid of additives. The morphology of the new membranes was dependent on the polymer, solvent/solvent system, and temperature of the casting solution, resulting in different morphologies (sponge‐like, finger‐like and macro‐voids and dense structures). Interestingly, pure solid **4** generated soft two‐layer membranes with a permeable porous layer supported on a dense layer. Membrane morphology was easily tailored when solvent and casting temperature were changed. Future investigations will include the testing of the membranes generated in this study to determine their suitable application. The cellulose acetate‐based membrane showed the highest thermal degradation when manufactured using pure **4**, whereas both polyethersulfone and polysulfone membranes were more thermally stable when cast from a solution containing Cg−Cy and pure **4** with small difference between the two solvents. However, polyimide membranes showed a high degradation temperature (over 600 °C) when **3** or Cg−Cy are applied as solvents. In conclusions, the morphology and thermal stability of the filtration membranes were easily tailored when the polymer, solvent and temperature were changed.

Regarding the biocatalytic synthesis reaction, if compared with Cyrene and other previously used solvents such as OMEs and DPE, **4** results as a very promising candidate for the enzymatic synthesis of high molecular weight aliphatic polyesters. Cygnet 0.0 performed very well in the synthesis of bulkier aliphatic‐aromatic FDCA‐based oligomers that can be further derivatized in a 2nd reaction step to yield useful surfactants or be used as plasticizers.

The enzymatic reactions performed in the less polar 4 led to polymers having a higher molecular weight than when using **3** or the Cg−Cy mixture. Cyrene has formed intra‐ and trans‐chain ketals with the subsequent loss of the polymer's hydroxy functionality when used in enzymatic transesterification reactions; Cg−Cy blend has had the same effect but in a lesser extent. **4** on the other hand had no detectable side reactions.

There is a great deal of related work that could be performed in the area of Cygnets and other **4** derivatives. Hansen Solubility Parameters in Practice software showed that other Cg−Cy mixtures could have the potential to be used as solvent systems in filtration membranes replacing the toxic polar aprotic solvents currently used, and potentially extend their use in other applications, where the need of replacing NMP or DMF is vital. However, the different Cg−Cy mixtures need further characterization, particularly with respect to their physical properties, e. g., viscosity, density, melting and boiling points. Performing these characterizing measurements could better inform choices about potential applications for these solvents.

## Experimental Section

### Chemicals and materials

The solvent Cyrene with a purity of 99.5 % was supplied by Circa Group., UK. The flakes of Ultrason® E3020 P Polyethersulfone (PES) of 55,000 Da were obtained from INGE.BASF, Germany. Cellulose acetate (CA) with *M*_W_≈50000 and polysulfone (PSf) pellets with *M*_W_≈35,000 were purchased from Sigma Aldrich (Merck). Polyimide (PI) of 588.616 (g/mol) was purchased from Fisher Scientific. All chemicals were used without any further purification. Deionized water (DI) was provided in‐house by the lab using an ELGA CENTRA® system. Cg−Cy in situ was produced in house for easy preparation of membranes. A 50 wt % Cg−Cy was produced by blending liquid **3** and pure **4** for polycondensation reactions. *Candida antarctica* lipase B (CaLB) immobilized onto a microporous resin was purchased from Sigma‐Aldrich and freeze dried before use (measured synthetic activity 9734 U/g PLU assay). All chemicals and solvents used for the biocatalytic synthesis work were used as received if not otherwise specified.

### Synthesis of Cygnet 0.0

**Figure 7:**
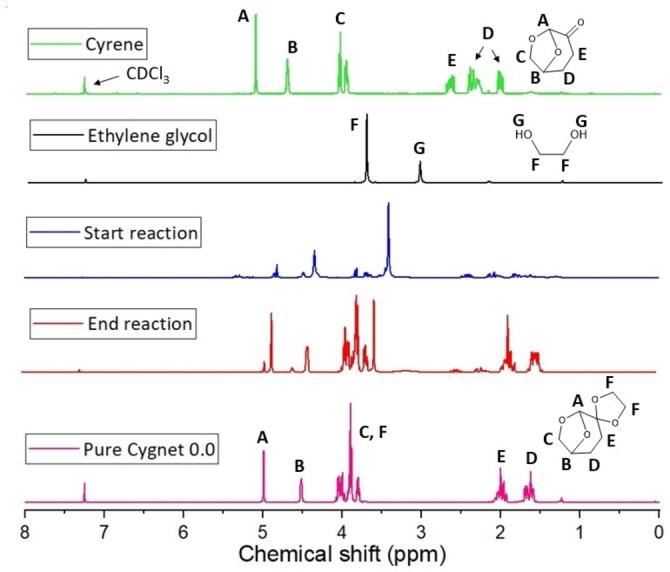
^1^H NMR spectra of Cygnet 0.0 and its starting materials (Cyrene and ethylene glycol) at the beginning and end of synthesis.

Cygnet 0.0 (**4**) was previously obtained from the reaction between Cyrene (**3**) and ethylene glycol at 100 °C, in the presence of an acidic catalyst (KSF200).^[21]^ The reagents were refluxed in toluene for 24 h and the recrystallization was done using an excess of ethylene glycol. In this study,

Cyrene (0.12 mol) and ethylene glycol (0.21 mol) were added in a round bottom flask with 0.75 g acid catalyst (KSF200). The mixture was heated under stirring to 100 °C for 1 h, after which the mixture was cooled to room temperature and the catalyst removed by vacuum filtration. Magnesium sulfate was added to remove any water traces, and the mixture filtrate once more. The solution was stored in the fridge overnight. **4** was recrystallized from ethanol, aligning well with green chemistry principles and no toluene was employed in the mechanism.^[98]^ The filtrate containing ethylene carbonate and ethanol is separate by removing the ethanol first, by using a rotary evaporator. The catalyst was washed and reactivated by carbonization at 200 °C for 3 h. The dramatic improvement in efficiency and ease of **4** synthesis will enable easier experimentation with the properties and applications of **4**. A yield of 85 % of pure Cygnet 0.0 was obtained. ^1^H NMR spectra of Cygnet 0.0 and its starting materials are shown in Figure 7.

### In situ synthesis of Cg−Cy

**Figure 8:**
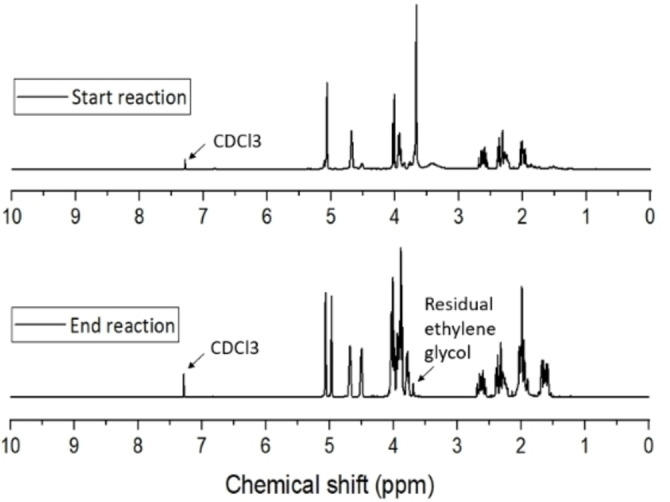
^1^H NMR spectra of Cg−Cy synthesis at starting and ending points.

Because **3** is the precursor for **4** (Figure 1), Cg−Cy blends could be conveniently synthesized by using ethylene glycol as limiting reagent (0.08 mol ethylene glycol and 0.12 mol Cyrene). Removal of water with a drying agent, followed by filtration to remove drying agent and catalyst, presents an attractive single‐step synthetic option, which requires no further purification. Residual ethylene glycol is detected by ^1^H NMR spectroscopy (Figure 8), which could be eliminated by extending the reaction time.

### Membranes’ fabrication

**Figure 9:**
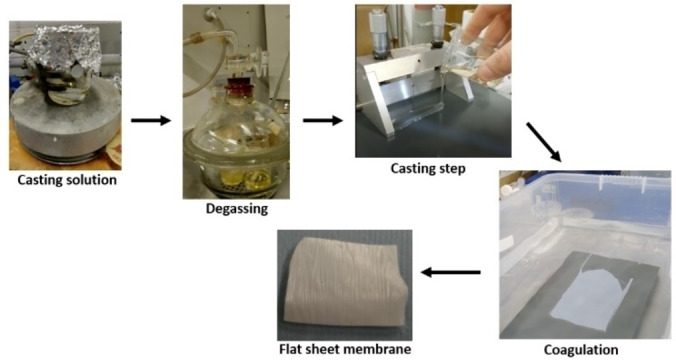
Illustration of the phase‐inversion technique used to cast filtration membranes.

In this study, the filtration membranes were fabricated from both hot (100 °C) and cold (RT) casting gels of four different polymers (PES, PSf, CA and PI) and three solvents/mixture of solvents (**3**, **4** and 50 wt % Cg−Cy), using a nonsolvent phase inversion technique (NIPS; Figure 9):

An amount of 15 % of each polymer was immersed in 85 % solvent and heated up to 100 °C for 4–6 h. Each polymer solution was cast at ambient temperature onto a glass plate using a steel blade for a thickness of 200 μm. The glass plate with the casting film was submerged in a coagulation bath containing deionized water at RT, where the polymers precipitated and formed stable membranes. The produced membranes were then washed distilled water to remove any residual solvent and stored in deionized water until further use. PI membranes were cast in water and stored in isopropanol.

The hot casting was possible due to keeping a quartz glass in oven at 100 °C for 20 min. A maximum of 3 min and a loss of few degrees were taken in account from the moment the quartz plate is taken from the oven to the moment the casting gel is placed onto it and the film cast.

### Biocatalytic polycondensation reactions

The biocatalytic polycondensation reactions were performed as previously reported with some modifications.[^22^, ^72^] Briefly, 8×10^−4^ mol of diester (0.2 M) and the equivalent molar amount of the selected diol (diester/diol ratio=1 : 1) were added together with 4 mL of solvent in a 25‐mL round bottomed flask and stirred at 85 °C until complete melting was obtained. In total, 10 wt % (calculated on the total amount of the monomers) of iCaLB was then added and the reaction was run for 6 h at 1000 mbar. A vacuum of 20 mbar was subsequently applied for an additional 90 h while maintaining the reaction *T* at 85 °C. A suitable solvent was added to the reaction mixture to solubilize the polymer product and the biocatalyst was filtered off. The solvent was then removed *via* rotary evaporation. The polymer‐solvent mixture was subsequently crashed out in ice‐cold methanol achieving precipitation of the products. Three methanol washing steps were subsequently performed to remove the residual reaction solvent traces. The reactions led to white powdery polymerization products. All reactions were carried out in duplicate.

### Characterization

Hansen Solubility Parameters was used in this project to predict solubility of PES in different solvents by mapping the dispersion (*δ*_D_), dipolarity (*δ*_P_) and hydrogen bonding ability (*δ*_H_) in a three‐dimensional Hansen space, using 5^th^ edition 5.0.03 of HSPiP software.

KAT parameters describe solvent polarity based on 3 parameters: hydrogen bond (HBD) ability (α), hydrogen bond accepting (HBA) ability (*β*) and a combination of dipolarity and polarizability (π*). π* is used to measure the ability of a solvent to stabilize a dipole or a neighboring charge by the function of nonspecific dielectric interactions.^[99]^ When all 3 parameters are used in a linear solvation energy relationship (LSER), they can explain a large number of solvent phenomena.^[100]^

ATR‐FTIR spectra were recorded on a PerkinElmer Spectrum 400 FT‐IR/FT‐NIR Spectrometer with transmittance peaks in 4000–650 cm^−1^ region, with rapid scanning (4 scans) and resolution 4 cm^−1^ at room temperature.

Scanning Electron Microscopy (SEM) images were taken on a JEOL JSM‐6490LV, at 8 kV from Bioscience Technology Facility, Biology Department, University of York. The flat sheet membranes were frozen and fractured in liquid nitrogen. They were then coated in an Au/Pd film and further used to determine the morphology and structure of the membranes.

Pure water permeability tests were assessed by a dead‐end filtration cell (effective membrane area=14.6 cm^2^, HP 4750, Sterlitech, USA) and the pure water flux(F) were measured by using Equation 1:

(1) $$F=\frac{V}{A\cdot t}$$

Where F (L ⋅ m^−2^ ⋅ h^−1^) is pure water flux, V (L) represents the pure water volume through the membrane during certain time duration,A (m^2^) is the effective area of the membrane being tested and t (h) is the operation time. All membrane samples were tested at room temperature and constant 300 rpm stirring. At the beginning of each test, the filtration cell was pressurized at 1 bar until stable flux was attained, then the pressure was increased to 5 bar and operated for 30 min. It is necessary to point out that each data is the average of at least three pieces of membranes to ensure the accuracy and validity of the data.

The dynamic viscosity of **3** and Cg−Cy were analyzed using a Malvern Kinexus pro+ rotational rheometer with a 40 mm diameter 4° angle cone over a 61 mm plate (CP4/40 SR2013 SS: PL61 ST S1540 SS). 1 mL solvent as used for each test and in triplicate. The software used to measure the viscosity vs. temperature was „Single frequency strain‐controlled temperature ramp“: ramp rate 1 °C, Start temperature 10 °C, end temperature 50 °C, Final temperature 25 °C, Frequency 1 Hz, Sampling interval 0.00:00:02.

^1^H NMR spectroscopy analyses were performed on a JEOL JNM‐ECS400 A spectrometer at a frequency of 400 MHz for ^1^H. CDCl_3_ was used as NMR solvent for all synthesized polymers.

Gel permeation chromatography was carried out at 30 °C on an Agilent Technologies HPLC System (Agilent Technologies 1260 Infinity) connected to a 17369 6.0 mm ID×40 mm L HHR−H, 5 μm Guard column and a 18055 7.8 mm ID×300 mm L GMHHR−N, 5 μm TSKgel liquid chromatography column (Tosoh Bioscience, Tessenderlo, Belgium) using 1 mL min^−1^ CHCl_3_ as mobile phase. An Agilent Technologies G1362 A refractive index detector was employed for detection. The molecular weights of the polymers were calculated using linear polystyrene calibration standards.

MALDI‐TOF MS analysis were carried out by using a Bruker Solarix‐XR FTICR mass spectrometer and the relative software package for the acquisition and the processing of the data. An acceleration voltage of 25 kV, using DCTB as matrix and KTFA as ionization agent were used. 10 μL of polymer solution were mixed with 10 μL of matrix solution (40 mg mL^−1^ DCTB in THF) and 3 μL of KTFA (5 mg mL^−1^). In total, 0.3 μL of the mixture were applied on the plate. The measurement was conducted in positive mode with the detector set in reflector mode.

DSC experiments were performed on a TA Instruments Q2000 DSC under an inert gas atmosphere (N_2_). The used heating and cooling rates were set to 5 °C over the *T* range of −60–200 °C. Sample mass was 5 mg for all analyzed samples. The *T*_c_ values were reported from the first cooling while the *T*_m_ values were reported from the second heating scan.

Thermogravimetric analysis (TGA) was performed on a PL Thermal Sciences STA 625 thermal analyzer. 10 mg of accurately weighed sample in an aluminum sample cup was placed into the furnace with a N_2_ flow of 100 mL min^−1^ and heated from room temperature to 625 °C at a heating rate of 10 °C min^−1^. From the TGA profiles the temperatures at 10 % and 50 % mass loss (*T*_d10_ and *T*_d50_, respectively) were determined.

### Enzymatic synthesis assay

The synthetic enzymatic activity was assayed using the propyl laurate assay as previously reported by Schilke and Kelly.^[101]^

## Conflict of interest

The authors declare no conflict of interest.

## Supporting information

As a service to our authors and readers, this journal provides supporting information supplied by the authors. Such materials are peer reviewed and may be re‐organized for online delivery, but are not copy‐edited or typeset. Technical support issues arising from supporting information (other than missing files) should be addressed to the authors.

Supporting Information
