# Supplementary material for: Polymer Chemistry Applications of Cyrene and its Derivative Cygnet 0.0 as Safer Replacements for Polar Aprotic Solvents
Source: ChemSusChem. 2021 Jul 16;14(16):3367–81. doi: 10.1002/cssc.202101125 (PMC8457101; doi:10.1002/cssc.202101125)
Supplement: Supplementary file 1 — Supporting Information [file CSSC-14-3367-s001.pdf]

ChemSusChem

Supporting Information

Polymer Chemistry Applications of Cyrene and its Derivative Cygnet 0.0 as Safer Replacements for Polar Aprotic Solvents

Roxana A. Milescu, Anna Zhenova, Marco Vastano, Richard Gammons, Shiliang Lin, Cher Hon Lau, James H. Clark, Con R. McElroy,* and Alessandro Pellis*© 2021 The Authors. ChemSusChem published by Wiley-VCH GmbH. This is an open access article under the terms of the Creative Commons Attribution License, which permits use, distribution and reproduction in any medium, provided the original work is properly cited.

Contents **Page**

Contents	Page
Hansen solubility parameters for the recommended solvent non/tested for dissolution of polyethersulfone (PES3020)	S2
Thermogravimetric (TGA) and differential scanning calorimetry (DSC) analyses of the aliphatic polymers synthesized in the various solvents using dimethyl adipate as the diester.	S3
The mixtures of Cygnet 0.0 in Cyrene (10-90 wt%) and pure Cygnet 0.0	S3
The dynamic viscosity of Cyrene (in black) and Cg-Cy 50-50 wt% solution (in red).	S4
The thermogravimetric (TGA) (a) and differential thermogravimetric (DTG) (b) spectra of CA (1), PES (2), PSf (3) and PI membranes (4); and the solvents Cyrene and Cygnet 0.0 (5)	S5
MALDI analysis of the polymer synthesized from DMA and BDO in Cyrene	S5
^{1}H-NMR spectra of poly(1,4-butylene adipate) synthesized in Cyrene.	S6
MALDI analysis of the polymer synthesized from DMA and ODO in Cyrene.	S6
^{1}H-NMR spectra of poly(1,8-octylene adipate) synthesized in Cyrene.	S7
^{1}H-NMR spectra of poly(1,4-butylene adipate) synthesized in the 50% Cy:Cg mixture.	S7
^{1}H-NMR spectra of poly(1,8-octylene adipate) synthesized in the 50% Cg:Cy mixture.	S8
MALDI analysis of the polymer synthesized from DMA and BDO in Cygnet.	S8
^{1}H-NMR spectra of poly(1,4-butylene adipate) synthesized in Cygnet 0.0.	S9
MALDI analysis of the polymer synthesized from DMA and ODO in Cygnet.	S9
^{1}H-NMR spectra of poly(1,8-octylene adipate) synthesized in Cygnet 0.0.	S10
TGA analysis of the polymers synthesized from DMA and ODO in the various organic solvents considered for the enzymatic synthesis reactions in the present work	S10
^{1}H-NMR spectra of the reaction’s blank	S11
^{13}C-NMR spectra of the polymers	S11

Table S1. Hansen solubility parameters for the recommended solvent non/tested for dissolution of polyethersulfone (PES3020).

Solvent or mixture of solvents	δ_D	δ_P	δ_H	Score	RED
40%Cygnet	18.7	10.7	7	-	0.418
30%Cygnet	18.7	11.1	7	-	0.431
50%Cygnet	18.6	10.3	7	-	0.433
20%Cygnet	18.8	11.6	7.1	-	0.435
60%Cygnet	18.5	9.9	7	-	0.454
70%Cygnet	18.5	9.5	7	-	0.456
10%Cygnet	18.8	12	7.1	-	0.458
Cyrene	18.9	12.4	7.1	1	0.467
80%Cygnet	18.4	9	6.9	-	0.488
90%Cygnet	18.4	8.6	6.9	-	0.5
Cygnet 0.0	18.3	8.2	6.9	-	0.538
N-Methyl-2-Pyrrolidone (NMP)	18	12.3	7.2	1	0.639
Methylene Dichloride (Dichloromethane)	17	7.3	7.1	-	0.862
N,N-Dimethyl Acetamide	16.8	11.5	9.4	1	0.934
Dimethyl Sulfate	16.5	13	7	5	0.992
Tetrahydrofuran (THF)	16.8	5.7	8	5	0.998
Dimethyl Sulfoxide (DMSO)	18.4	16.4	10.2	1	0.998
Propylene Carbonate	20	18	4.1	4	1.001
Dimethyl Formamide (DMF)	17.4	13.7	11.3	1	1.008
Chloroform	17.8	3.1	5.7	4	1.014
1,1-Dichloroethane	16.5	7.8	3	1	1.041
Benzyl Alcohol	18.4	6.3	13.7	4	1.055
Sulfolane (Tetramethylene Sulfone)	17.8	17.4	8.7	5	1.106
2-Methylfuran	17.3	2.8	7.4	5	1.116
Acetone	15.5	10.4	7	4	1.159
Ethyl Acetate	15.8	5.3	7.2	5	1.216
Dimethyl Carbonate	15.5	8.6	9.7	5	1.224
TMTHF	15.5	8.6	9.7	5	1.224
Toluene	18	1.4	2	5	1.268
p-Xylene	17.8	1	3.1	5	1.28
n-Propyl Acetate	15.3	4.3	7.6	5	1.378
Acetonitrile	15.3	18	6.1	5	1.536
1-Butanol	16	5.7	15.8	5	1.584
Propionic Acid	14.7	5.3	12.4	5	1.605
Cyclohexane	16.8	0	0.2	5	1.624
Diethyl Ether	14.5	2.9	4.6	5	1.633
2-Propanol	15.8	6.1	16.4	5	1.65
1-Propanol	16	6.8	17.4	5	1.686

Heptane	15.3	0	0	5	1.843
Ethanol	15.8	8.8	19.4	5	1.867
Hexane	14.9	0	0	5	1.906
Methanol	14.7	12.3	22.3	5	2.308
Water	15.5	16	42.3	5	4.422

Table 2. Thermogravimetric (TGA) and differential scanning calorimetry (DSC) analyses of the aliphatic polymers synthesized in the various solvents using dimethyl adipate as the diester.

		TGA			DSC		
**Solvent**	**Diol**	**T_{d10} [°C]**	**T_{d50} [°C]**	**Residue at 625 C [%]**	**T_c [°C]**	**T_{m1} [°C]**	**T_{m2} [°C]**
Cyrene	BDO	359	391	10	26	43	-
	ODO	369	399	2	46	61	-
Cg:Cy	BDO	361	393	3	32	48	50
	ODO	372	400	6	42	61	-
Cygnet	BDO	364	394	2	35	49	55
	ODO	376	402	3	51	66	-

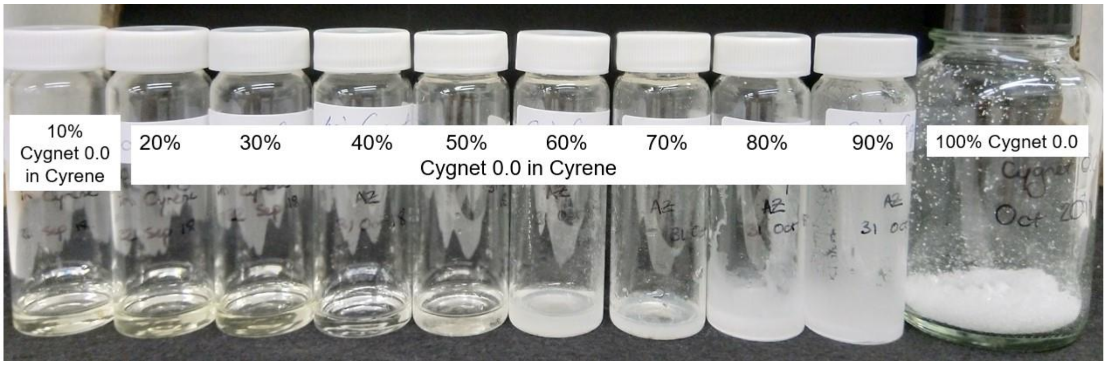

Figure S1. The mixtures of Cygnet 0.0 in Cyrene (10-90 wt%) and pure Cygnet 0.0

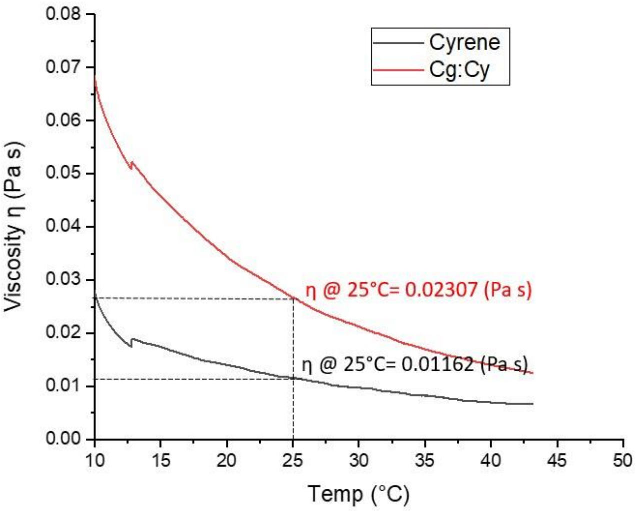

Figure S2. The dynamic viscosity of Cyrene (in black) and Cg-Cy 50-50 wt% solution (in red).

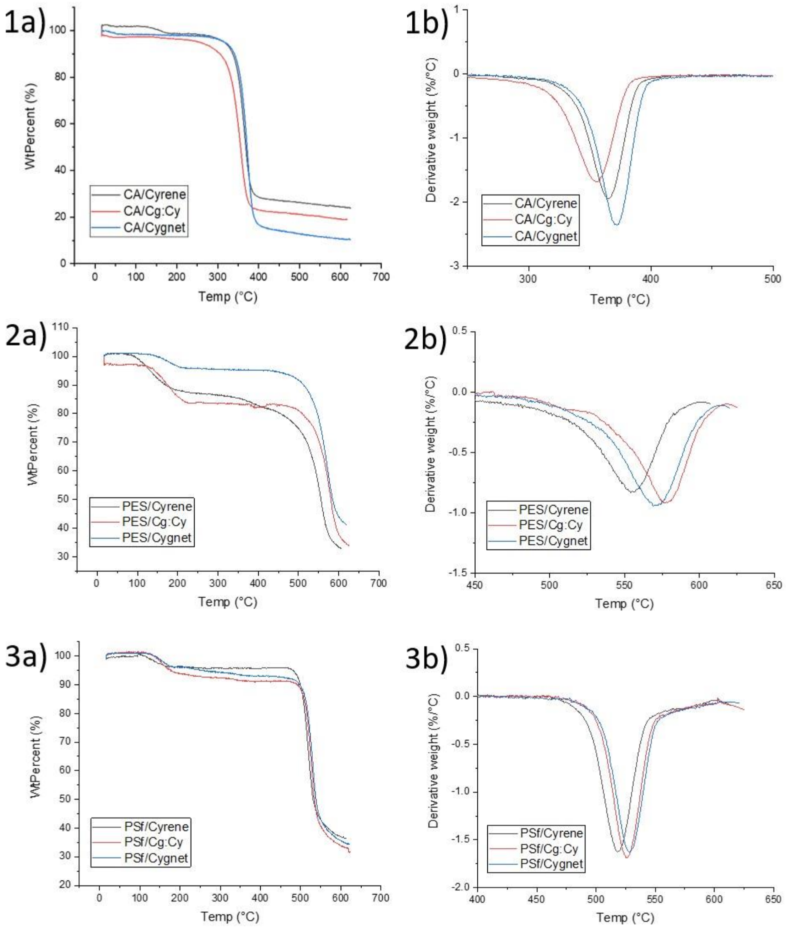

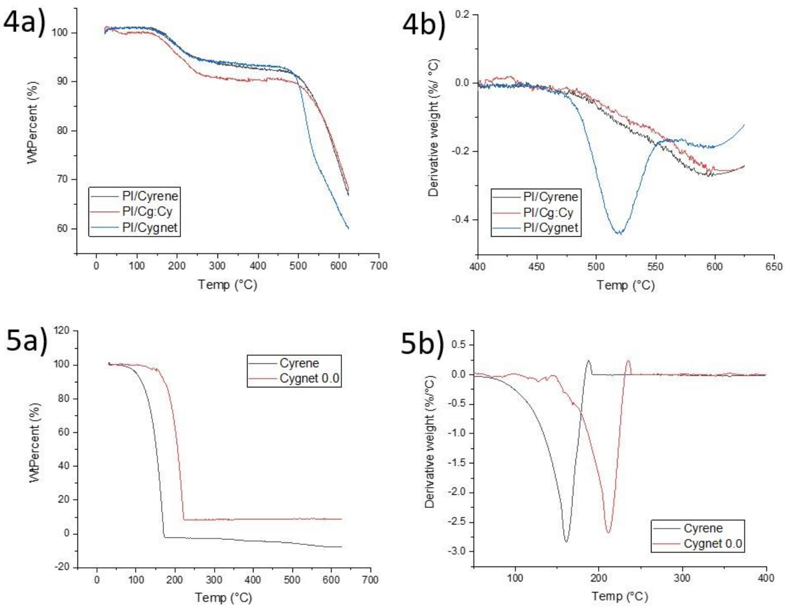

Figure S3. The thermogravimetric (TGA) (a) and differential thermogravimetric (DTG) (b) spectra of CA (1), PES (2), PSf (3) and PI membranes (4); and the solvents Cyrene and Cygnet 0.0 (5)

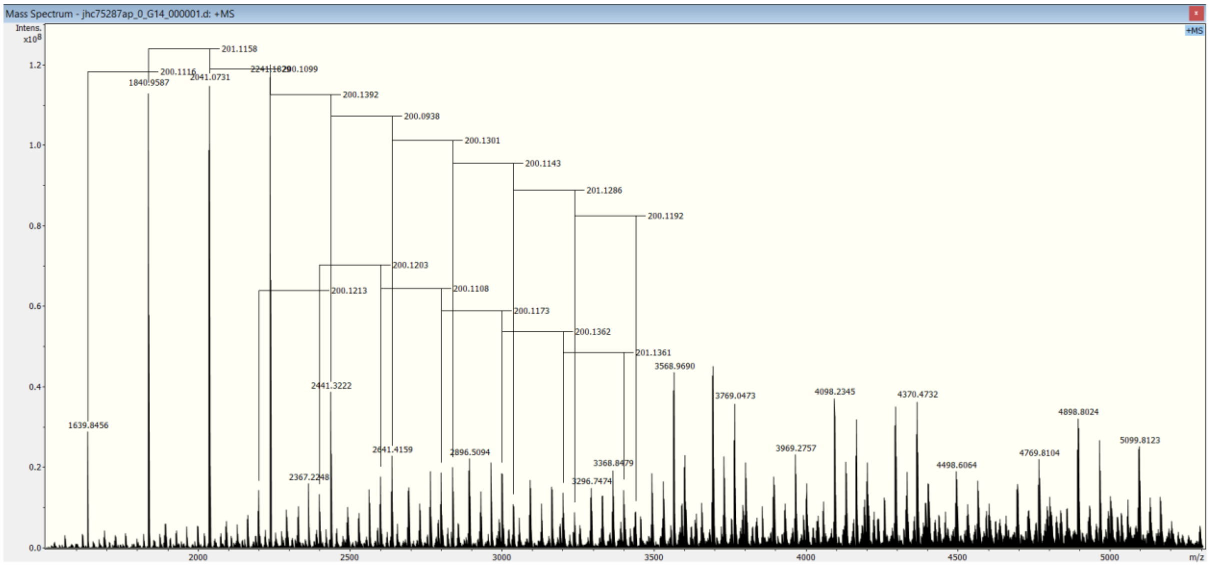

Figure S4. MALDI analysis of the polymer synthesized from DMA and BDO in Cyrene.

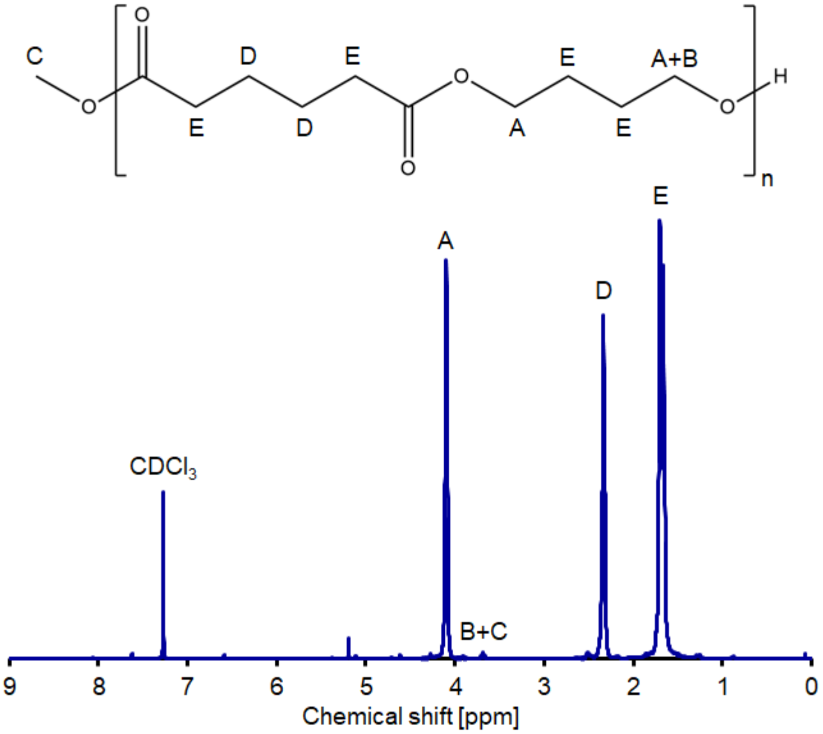

Figure S5. ^{1}H-NMR spectra of poly(1,4-butylene adipate) synthesized in Cyrene.

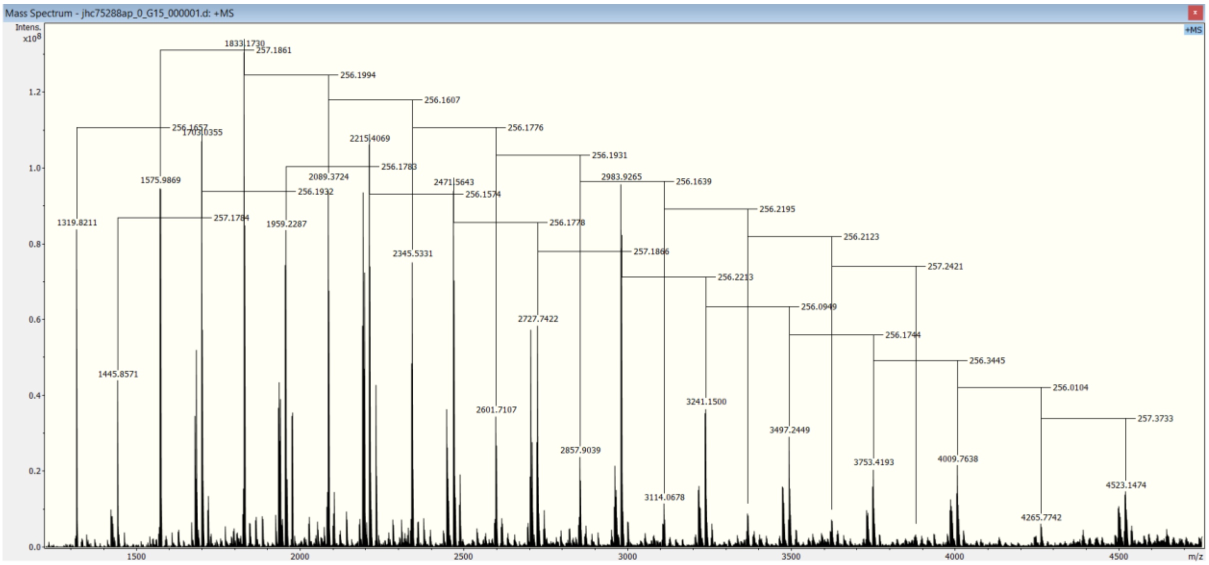

Figure S6. MALDI analysis of the polymer synthesized from DMA and ODO in Cyrene.

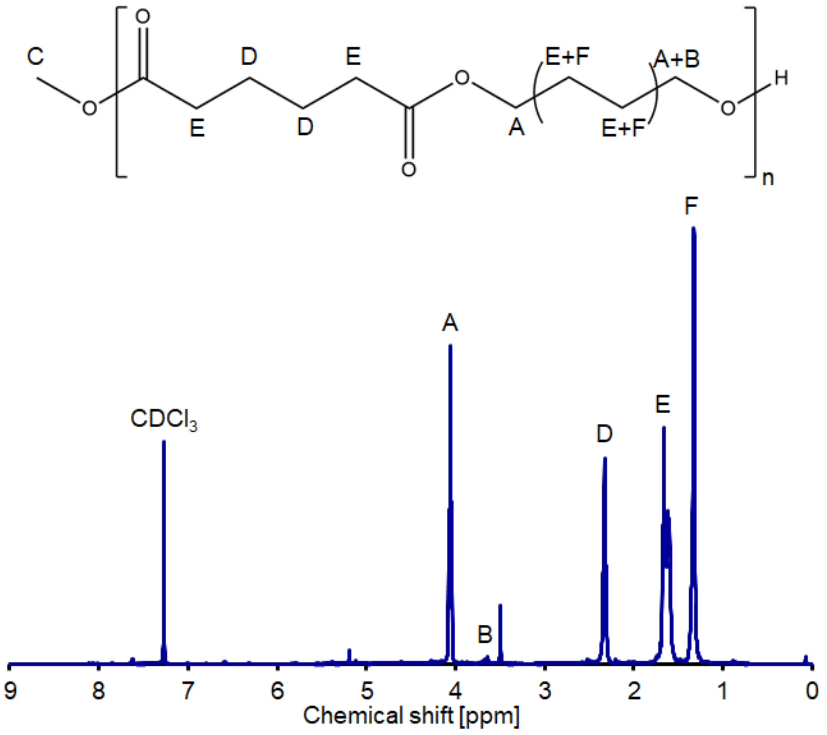

Figure S7. ^{1}H-NMR spectra of poly(1,8-octylene adipate) synthesized in Cyrene.

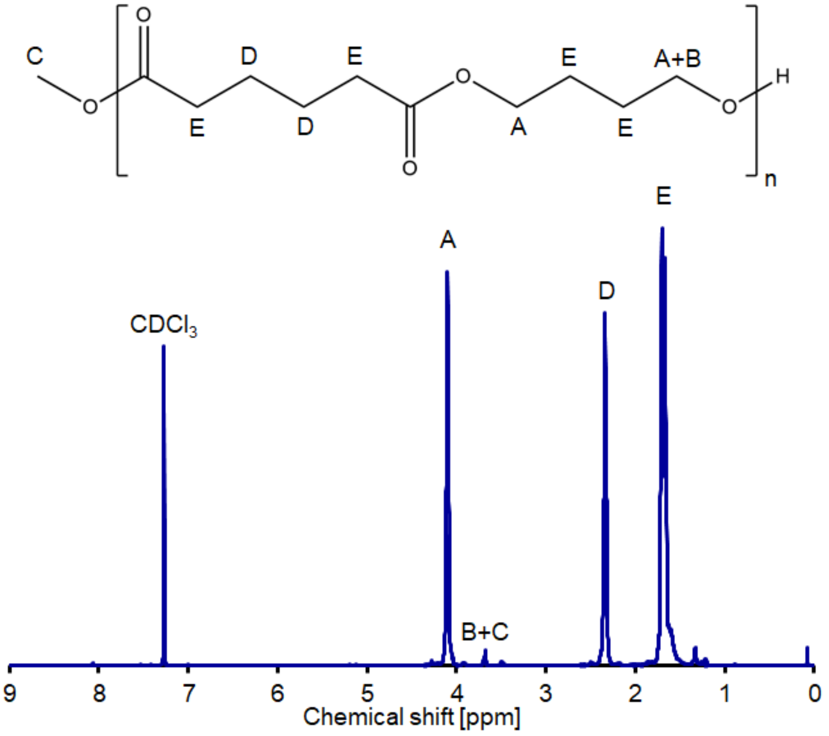

Figure S8. ^{1}H-NMR spectra of poly(1,4-butylene adipate) synthesized in the 50% Cy:Cg mixture.

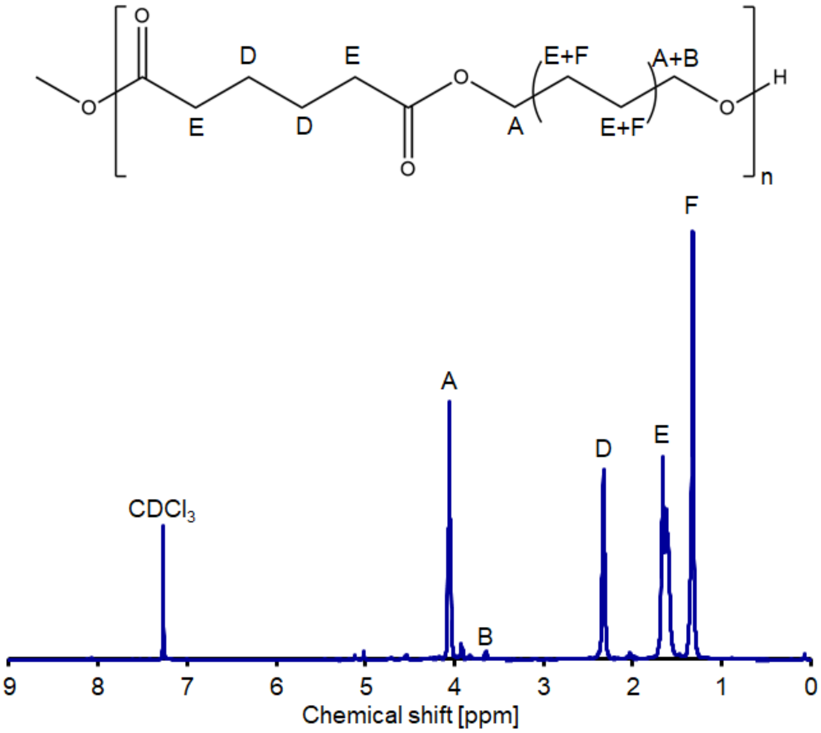

Figure S9. ^{1}H-NMR spectra of poly(1,8-octylene adipate) synthesized in the 50% Cg:Cy mixture.

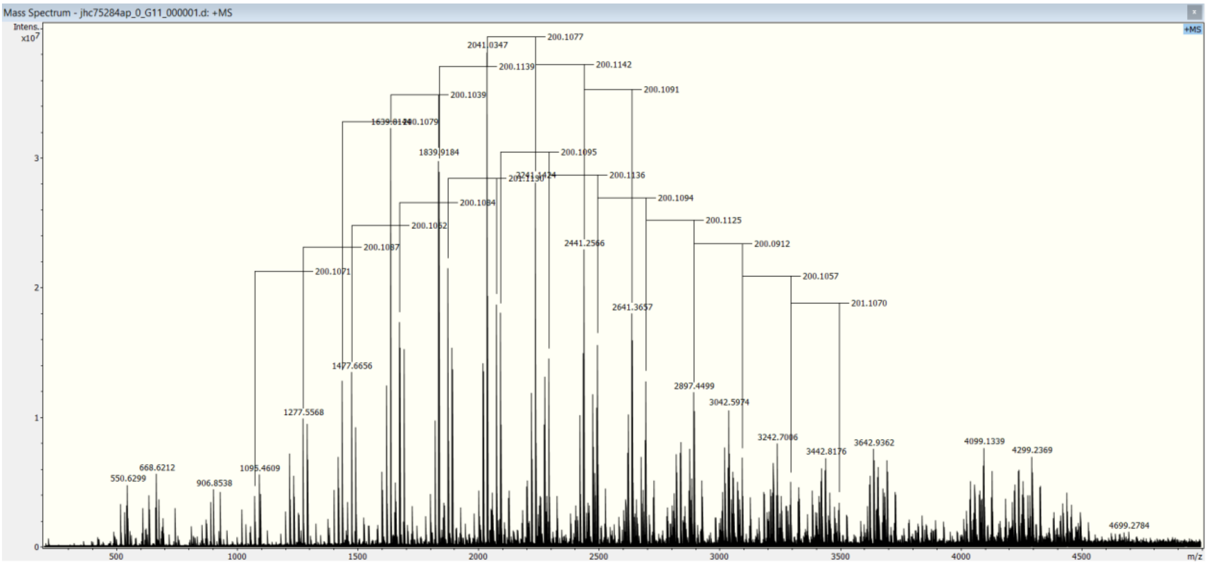

Figure S10. MALDI analysis of the polymer synthesized from DMA and BDO in Cygnet.

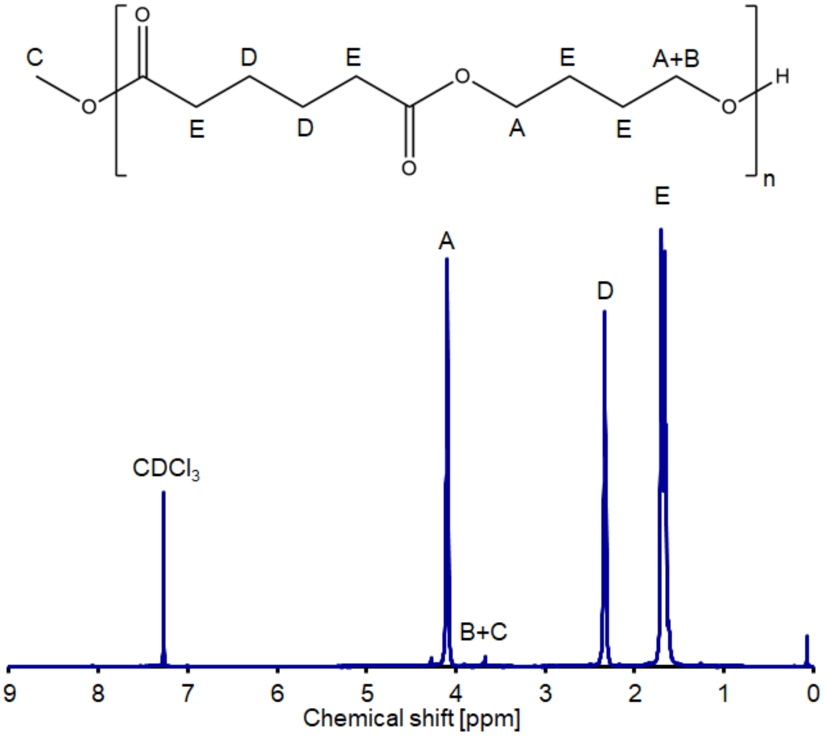

Figure S11. ^{1}H-NMR spectra of poly(1,4-butylene adipate) synthesized in Cygnet 0.0.

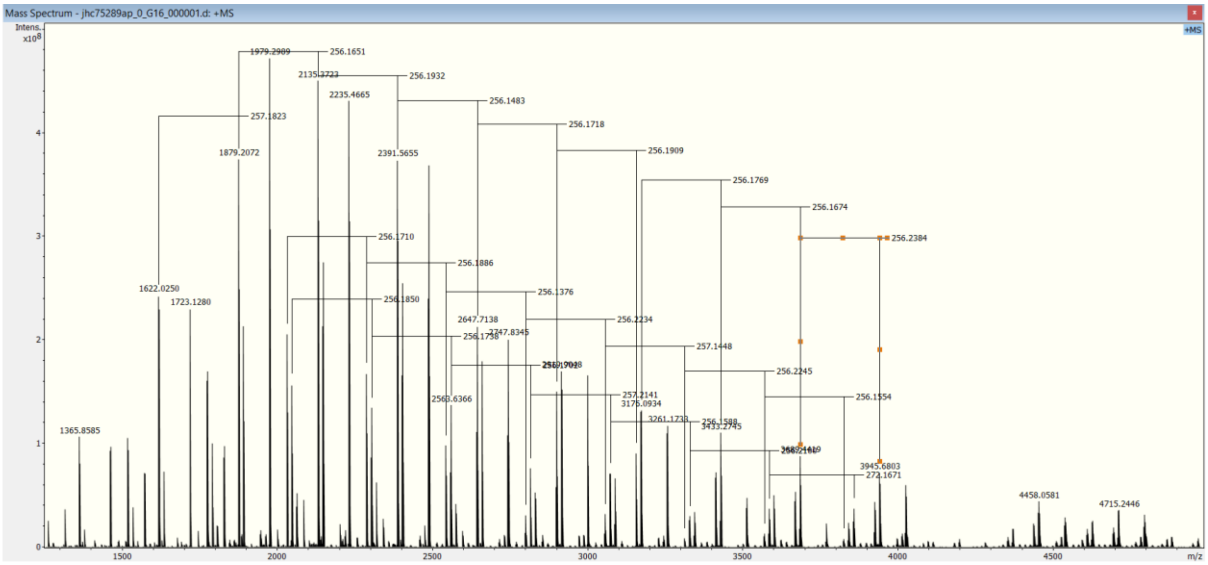

Figure S12. MALDI analysis of the polymer synthesized from DMA and ODO in Cygnet.

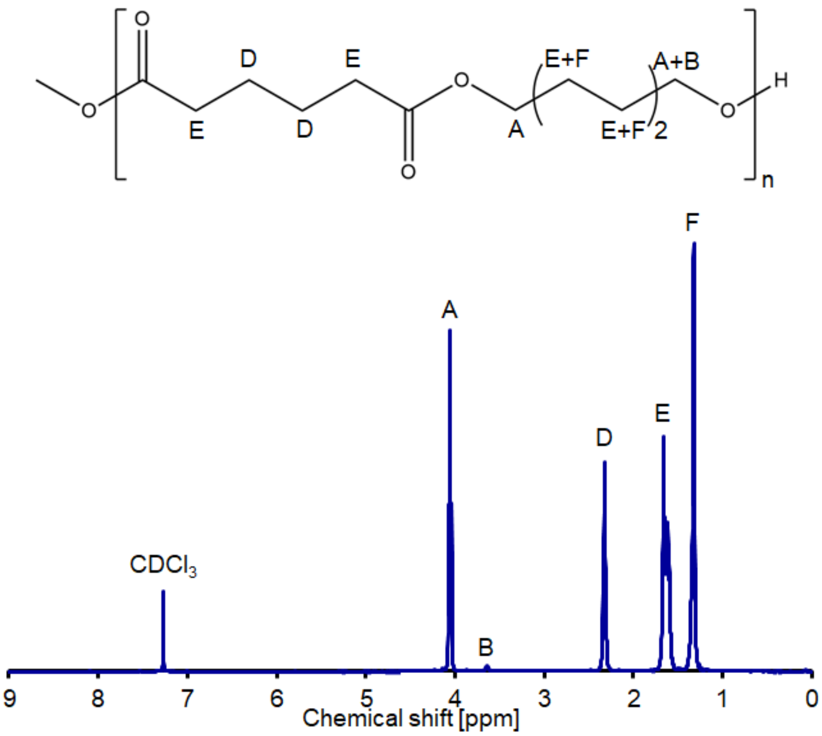

Figure S13. ^{1}H-NMR spectra of poly(1,8-octylene adipate) synthesized in Cygnet 0.0.

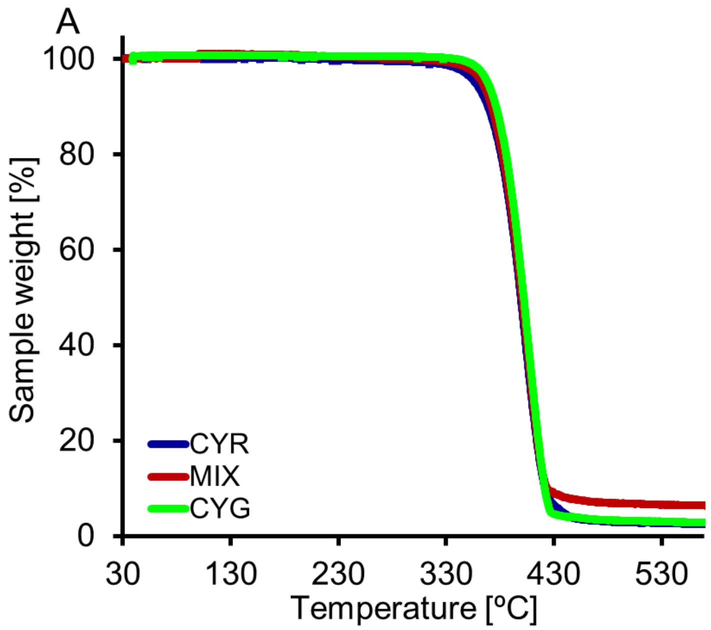

Figure S14. TGA analysis of the polymers synthesized from DMA and ODO in the various organic solvents considered for the enzymatic synthesis reactions in the present work.

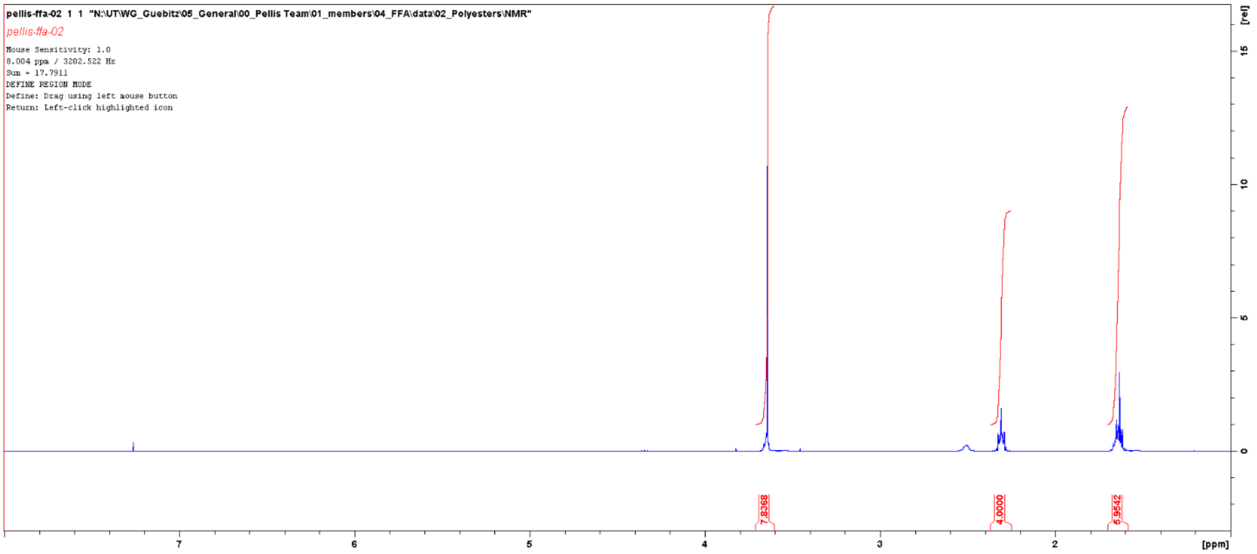

Figure S15. ^{1}H-NMR spectra of the dimethyl adipate + 1,4-butanediol reaction blank (monomers mixed at 85 C without the addition of the enzyme and the solvent).

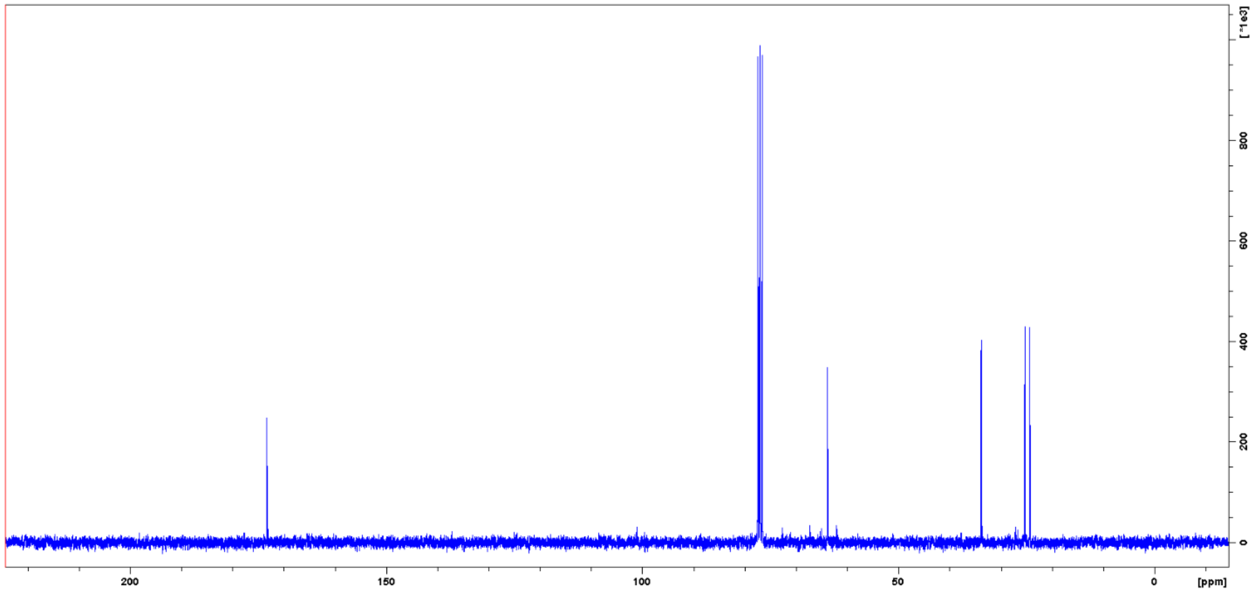

Figure S16. ^{13}C-NMR spectra of poly(1,4-butylene adipate) synthesized in Cygnet 0.0

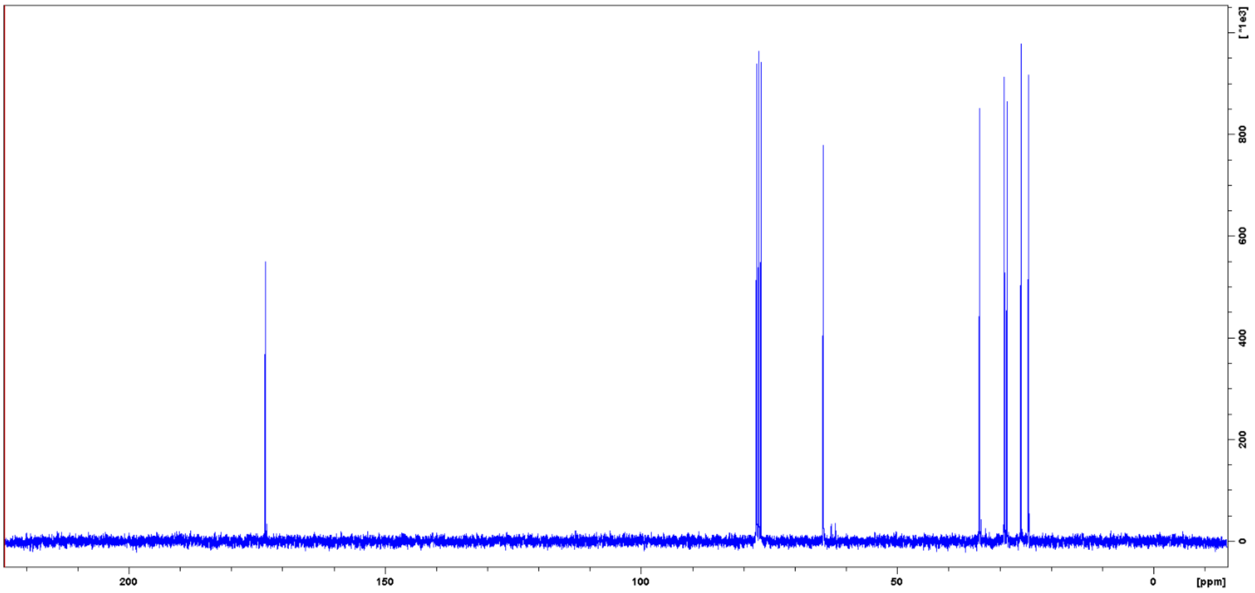

Figure S17. ^{13}C-NMR spectra of poly(1,8-octylene adipate) synthesized in Cygnet 0.0.
